# Paleomagnetic Evidence for Inverse Correspondence between the Relative Contribution of the Axial Dipole Field and CMB Heat Flux for the Past 270 Myr

**DOI:** 10.1038/s41598-018-36494-x

**Published:** 2019-01-22

**Authors:** Daniel Ribeiro Franco, Wellington Paulo de Oliveira, Felipe Barbosa Venâncio de Freitas, Diego Takahashi, Cosme Ferreira da Ponte Neto, Ian Muzy Camarão Peixoto

**Affiliations:** 1 Coordenação de Geofísica, Observatório Nacional, R. Gal. José Cristino, 77, 20921-400 Rio de Janeiro, RJ Brazil; 2 Instituto de Geociências, Universidade Federal Fluminense, Av. Milton Tavares de Souza, S/N, 24210-346 Niterói, RJ Brazil

**Keywords:** Core processes, Geodynamics, Palaeomagnetism

## Abstract

We provide an evaluation of the paleolatitudinal dependence of the paleosecular variation throughout the Paleozoic-Mesozoic transition – linked to the high geomagnetic reversal frequency interval Illawarra Hyperzone of Mixed Polarity (IHMP; ~266.7–228.7 Myr). Our findings were compared with those for intervals of distinctive geomagnetic reversal frequencies within the Phanerozoic. Our results for the IHMP were conducted through estimates of angular dispersion (*S*_*B*_) of virtual geomagnetic pole (VGP) data groups, taken from a high quality paleomagnetic database. Model G was fitted to these data, providing its shape parameters *a* and *b* (respectively related to the antisymmetric and symmetric harmonic terms for the time-average geomagnetic field). Results for the IHMP exhibited compatible patterns with two well-known intervals of higher reversal frequency – Jurassic and the last 5 Myr. A comparison of *b*/*a* ratio results – considered as an efficient indicator for the relative contribution of the axial dipole field – for the last 270 Myr, indicated an inverse correspondence with the relative core-mantle boundary (CMB) heat flux, according to recent discussions, clarifying the physical meaning of the Model G shape parameters *a* and *b*.

## Introduction

The phenomenological aspects of geodynamo that imply long-term changes for the geomagnetic field behavior have been an important subject of debate in literature^1–4^. Important progress toward a better understanding of the geodynamo has been made by means of more realistic numerical modelings, which better emulated geodynamic conditions throughout geologic eras^5–10^. Nevertheless, some of the long-standing questions refer to the Earth’s magnetic field (EMF) reversibility and its large-scale variations in average reversal rate are still a demand. It is well-known that the geomagnetic polarity timescale (GPTS) for the last 160 Myr indicates wide changings for the rate of geomagnetic reversals, reflecting the variable stability of geodynamo – from around 4-5 Myr^−1^, with an average duration for the polarity chrons of ~200 kyr for the past 15 Myr, reaching down to ~0.05 Myr^−1^ during the so-called 84–125 Myr Cretaceous Normal Superchron (CNS)^11–14^.

Although a stochastic contribution to the high variable geomagnetic reversal spectra cannot be ruled out^15,16^, there is important evidence for long-term modulations on the reversal rates by mantle convection^13,17–20^, which is plausible, taking into account the timescale differences between the shorter term, outer core convection and the GPTS – the latter being compatible to the mantle convection timescale^14,21^. Some authors (e.g., refs ^20–23^) suggest that such changes in reversal rate would be a result of spatial variability of the heat flux at the core-mantle boundary (CMB) throughout the Phanerozoic, although the connections between the geomagnetic reversal frequency and long-term mantle dynamics are still far from being completely clarified^16^.

Additionally, it has been discussed by some authors (e.g., refs ^9,24^) that the geodynamo exhibited more stability conditions (i.e. lower geomagnetic reversal rates) in periods when the main contribution to the geomagnetic field is given by the axial dipole field – which can be given by the antisymmetric spherical harmonic terms, as a solution for a field generated by a spherical geodynamo – in relation to the non-axial dipole contribution. Such conditions have been linked to ‘superchrons’ (~10^7^ yr, single geomagnetic polarity periods), as discussed by Biggin *et al*. (ref. ^25^) for the CNS, and for the 262–318 Myr Permian-Carboniferous Reversed Superchron (PCRS; ref. ^26^). Conversely, a lower dipolar contribution was reported for intervals of higher reversal frequency, such as the Jurassic^25,27^ and the last 5 Ma^28^.

Such information can be acquired by evaluations of the ancient geomagnetic field through analyses of paleosecular variation (PSV), related to the spatio-temporal variability in both direction and intensity of the EMF^8,22^. It provides an independent way of investigating the EMF evolution through geological time, hence it is adequate for assessing information on the time-averaged field, and its dipolar and non-dipolar contributors^4,25,29,30^. The PSV is commonly obtained by the angular dispersion (*S*) of virtual geomagnetic poles (VGPs) datasets, given by:

1 $$S=\sqrt{\frac{1}{N-1}\sum _{i=1}^{N}{{\rm{\Delta }}}_{i}^{2}}$$

where *N* and Δ_i_ are, respectively, the number of VGPs and the angular difference between the ith VGP and the mean VGP. A phenomenological model that has been successfully employed for evaluation of *S* – which demonstrated a clear relation between reversal frequency and the latitudinal dependence of VGP dispersions^24^, was proposed by McFadden *et al*. (ref. ^31^). This approach (Model G) considers that the VGP angular dispersion results from the contribution of two independent “families” – dipole (*S*_*D*_) and quadrupole (*S*_*Q*_) families, which are respectively related to odd and even *l*–*m* spherical harmonic terms (i.e., asymmetric and symmetric around the equator region):

2 $$S(\lambda )=\sqrt{{({S}_{Q})}^{2}+{({S}_{D})}^{2}}=\sqrt{{a}^{2}+{(b\lambda )}^{2}}$$

where *λ* is the paleolatitude, and *a* and *b* are the Model G shape parameters (which are empirical constants that are respectively related to the quadrupole (symmetric) and dipole (antisymmetric) families of the field).

From hemispherically averaged VGP dispersion datasets carried out from 0–5 Ma lava flows, McFadden *et al*. (ref. ^27^) reported a possible correspondence for the past 160 Myr between the reversal frequency and the ratio *b/a* – which provides an empirical evaluation of the relative contribution of antisymmetric (*b*) to symmetric (*a*) harmonics terms of the geodynamo. Furthermore, Coe and Glatzmaier (ref. ^24^) reported by means of modeling simulations of the geodynamo that the symmetry of the time-averaged field – which can also be indicated by the ratio *b/a* – can be a better predictor of reversal frequency in comparison to the intensity evaluations.

Nevertheless, some important questions are still far from being completely elucidated about the extension of the large-scale variations for the reversal frequency, and its connections to the CMB heat flux fluctuations (linked to the long-term mantle dynamics) throughout the Phanerozoic. For instance, there are no reported discussions so far for:

(i) a possible lower contribution of the antisymmetric family for the high reversal rate interval known as Illawarra Hyperzone of Mixed Polarity (IHMP; ~266.7–228.7 Myr). The IHMP is characterized by a high mean geomagnetic reversal frequency (comprising tens of polarity reversal events from the end of PCRS (Late Permian) to the lowermost Triassic^32–34^), and is possibly related to some of the prominent geodynamic events that took place during the Paleozoic-Mesozoic transition^35,36^;
(ii) the extension of the original evaluation by means of *b*/*a* ratio as a function of reversal frequency proposed by McFadden *et al*. (ref. ^27^) and Coe and Glatzmaier (ref. ^24^) for Pre-Jurassic times, to achieve a better description of such behavior throughout the Phanerozoic;
(iii) comparisons about the mean CMB heat flux and the *b*/*a* ratio, in order to verify a possible correspondence between both factors.

In this work, we aim to address these points, in order to provide new information for the discussions that linked the long-term variations of the geomagnetic reversals, the geodynamo’s stability and the geodynamic processes throughout the Phanerozoic.

## Methods

### IHMP: selection criteria for the paleomagnetic database

In order to assess of the paleolatitudinal dependence of the paleosecular variation for the IHMP interval (~266.7–228.7 Myr), we conducted a pre-selection of paleomagnetic studies available in literature for this time interval, comprising of 112 works published between 1990 and 2018 based on igneous rocks. Such preliminary database research was carried out by means of academic search engines (e.g., *Web of Science* (https://www.webofknowledge.com/) and *Scopus* (https://www.scopus.com/home.uri)) and the IAGA’s Global Paleomagnetic Database (http://www.ngu.no/geodynamics/gpmdb/). Regarding the scarcity of studies based on highly sensitive magnetometers, which were often associated to low accuracy rock magnetism investigations, we did not consider datasets prior to 1990, according to similar procedures adopted by De Oliveira *et al*. (ref. ^26^).

From the preliminary dataset, we built the “final” paleomagnetic database by means of the following selection criteria: (1) all works that did not provide directional, characteristic remanent magnetization (ChRM) data per site and site coordinates, as well as at least ten sampling sites (N < 10) were ruled out; (2) preference was given to the selection of works which provide high-quality paleomagnetic poles in accordance to the Van der Voo (ref. ^37^) quality criteria; (3) the selected studies shall be related to level ≥ 4 of the GPMDB Demagcode procedure protocol^38,39^ as reliable analyses of VGP dispersion datasets can be prevented due to the employment of inadequate demagnetization procedures^40^; (4) only studies that succeeded in the recalculation of its paleomagnetic pole (s) and associated paleolatitude (s) by means of its ChRM directional data and site coordinates were considered. In order to remove spurious data that could be possibly related to eventual excursional fields or lightning occurrences that may influence the VGP angular dispersion, due to the size of the paleomagnetic datasets De Oliveira *et al*. (ref. ^26^), all selected paleomagnetic datasets were submitted to the Vandamme (ref. ^41^) iterative method. We ruled out the usage of a fixed cut-off angle regarding it could lead to overestimation (underestimation) of the angular standard deviation for low (high) latitudes Tauxe *et al*. (ref. ^42^).

The resulting paleomagnetic database from application of selection criteria #1 – #4 is constituted of 16 VGP datasets, provided by 12 paleomagnetic studies (which corresponds to ~14.3% of the pre-selected works), from igneous-based lithologies (Table 1; Supplementary Information Tables S1 and S2). However, as some of the datasets exhibit considerably high k-values (>200), we adopted an additional procedure to evaluate whether such corresponding VGP distributions represent adequate PSV samplings, by means of application of the Deenen *et al*. (ref. ^43^) criteria. It provides a N-dependent A95 envelope defined by a range of upper (A95_max_) and lower (A95_min_) limits, in which the observed A95 shall be within for a sufficient PSV sampling. As discussed by some authors (e.g., ref. ^43,44^), datasets that provide A95 > A95_max_ may contain additional scatter contributors, whereas A95 < A95_min_ could be considered as an indicator for an EMF spot-reading record. It was noticed that four of the select datasets (datasets # 2, 8, 10 and 15) provided A95 values that fall out of the A95_min_/A95_max_ range, and hence were not considered for the paleomagnetic data processing and the subsequent Model G curve fitting for the IHMP.

**Table 1:** Selected paleomagnetic database and its related statistical parameters.

Nr.	Study	Age (Myr)	Region of study	Rock type	DC	P	N_0_	N	D (°)	I (°)	k	λ_p_ (°N)	ϕ_p_ (°E)	A_95_ (°)	A_95 max_ (°)	A_95 min_ (°)	S (°)	S_B_ (°)	S_l_ (°)	S_u_ (°)	ΔS (°)	λ (°)
1	Kravchinsky *et al*. (2002)^A^	250.0 ± 1.6	Siberian platform (Russia)	Basalt flows	4	N	10	10	102.9	81.6	89.4	58.9	142.3	9.6	19.2	4.7	15.9	12.3	7.0	14.1	3.6	73.6
***2***	***Veselovskiy et al***. (***2012***)^***B***^	***245–232***	***Siberian platform*** (***Russia***)	***Doleritos***, ***dykes and trachyandesites***	***4***	***M***	***18***	***17***	***112***.***0***	***76***.***9***	***7***.***6***	***54***.***9***	***157***.***4***	***14***.***9***	***13***.***8***	***3***.***9***	***22***.***3***	***16***.***3***	***7***.***1***	***20***.***9***	***6***.***0***	***68***.***3***
3	Latyshev *et al*. (2018)	~250	Siberian traps (Russia)	Doleritos, basalt flows	5	N	35	35	93.9	78.8	75.7	56.3	138.7	5.1	8.7	2.9	16.6	15.9	12.7	18.4	0.7	68.3
4	Heunemann *et al*. (2004)^C^	~250	Siberian traps (Russia)	Lava flows	4	N	41	41	88.6	75.5	79.8	57.7	147.1	4.3	7.9	2.7	15.4	15.0	11.9	17.3	0.4	62.6
5	Pavlov *et al*. (2011)	~250	Siberian Traps-Kotui river valley (Russia)	Lava flows	4	M	70	69	120.0	74.7	77.0	49.4	141.0	3.4	5.7	2.2	15.2	13.5	11.3	15.3	1.7	61.2
6	Veselovskiy *et al*. (2012)^D^	250.0 ± 0.3	Siberian plataform (Russia)	Dykes	4	R	20	20	93.9	74.1	53.1	55.2	157.6	7.5	12.4	3.6	18.3	12.8	7.2	16.8	5.5	60.4
7	Gurevitch *et al*. (2004)	255.3 ± 5.3	Siberian traps (Russia)	Flood basalts	4	M	12	12	92.9	72.9	28.1	53.5	148.6	13.6	17.1	4.4	24.7	24.0	13.6	30.0	0.7	58.3
***8***	***Heunemann et al***. (***2004***)^***E***^	***~250***	***Siberian traps*** (***Russia***)	***Lava flows***	***4***	***N***	***14***	***14***	***22***.***0***	***68***.***3***	***234***.***8***	***68***.***8***	***230***.***2***	***4***.***1***	***15***.***6***	***4***.***2***	***8***.***3***	***7***.***5***	***5***.***1***	***9***.***0***	***0***.***8***	***51***.***5***
9	Kravchinsky *et al*. (2002)^F^	250.0 ± 1.6	Siberian platform (Russia)	Basalt flows	4	R	10	10	273.3	-64.1	201.5	40.0	176.5	5.1	19.2	4.8	8.4	6.5	3.1	8.1	1.9	45.9
***10***	***Heunemann et al***. (***2004***)^***G***^	***~251***	***Siberian traps*** (***Russia***)	***Lava flows***	***4***	***N***	***15***	***15***	***152***.***0***	***54***.***4***	***323***.***9***	***16***.***6***	***112***.***4***	***2***.***6***	***14***.***9***	***4***.***1***	***5***.***5***	***3***.***9***	***0***.***9***	***5***.***7***	***1***.***6***	***35***.***0***
11	Van der Voo *et al*. (1993)	251–260	Emeishan Basalts (China)	Basalts	4	N	10	10	25.7	-11.7	57.9	50.2	240.3	5.9	19.2	4.8	9.9	9.7	5.4	12.6	0.2	5.9
12	Yokoyama *et al*. (2014)	254.7 ± 2.5	Cratonic South America - Araguainha (Brazil)	Granite	5	R	27	26	357.5	-39.0	60.1	−84.1	330.2	3.9	10.5	3.2	9.6	8.8	5.9	10.9	0.8	−22.0
13	Tomezzoli *et al*. (2009)	240–260	Sierra Chica (Argentina)	Trachyandesitic pyroclastic flow, rhyolite	4	N	10	10	148.4	53.0	17.0	−64.4	17.0	14.6	19.2	4.8	23.8	23.2	12.3	27.0	0.6	−33.6
14	Miguez *et al*. (2016)	245–260	La Esperanza Plutono-Volcanic (Argentina)	Rhyolitic dykes	5	M	13	13	351.7	−59.0	28.6	−83.2	11.2	10.5	16.3	4.3	20.1	18.9	12.4	22.7	1.2	−39.8
***15***	***Domeier et al***. (***2011***)	***263***.***0 ± 1***.***6***	***Sierra Chica*** (***Argentina***)	***Ignimbrites***, ***tuffs***, ***trachyandesitic***	***5***	***M***	***35***	***33***	***170***.***5***	***60***.***0***	***3***.***5***	***−81***.***7***	***356***.***2***	***15***.***0***	***9***.***1***	***3***.***0***	***10***.***4***	***9***.***0***	***7***.***1***	***11***.***2***	***1***.***4***	***−40***.***8***
16	Belica *et al*. (2017)	~265	Sydney Basin (Australia)	Basalts and andesites	5	N	17	17	173.3	77.8	48.3	−56.9	154.8	9.1	13.8	3.9	20.2	17.5	12.7	18.4	2.7	−66.6
																					$$\overline{{\rm{\Delta }}S}$$ (°)	
																**Igneous rocks**					1.9	

DC: procedure protocol number *Demagcode*^38^; P: geomagnetic polarity (R: reverse; N: normal; M: mix); N_0_(N): Number of sites before (after) the application of variable cut-off angle^41^; D e I: ChRM declination, inclination, respectively; k: Fisher precision parameter; *λ*_*p*_ and *ϕ*_p_: latitude and longitude of paleomagnetic pole, respectively; *A*_95_: 95% confidence cone determined from the mean VGP distributions; *A*_95max_ and *A*_95min_: respectively, upper and lower limits of *A*_95_^43^; *S:* VGP dispersion; *S*_B_, *S*_*u*_ and *S*_*l*_: respectively, between-site VGP dispersion and its associated upper and lower 95 per cent confidence limits (obtained by the bootstrap method); ΔS: difference between *S* and *S*_*B*_; λ: paleolatitude; ^A^Aikhal; ^B^Udzha; ^C^Abagalakh; ^D^Delkan; ^E^Group C; ^F^Sytikanskaya; ^G^Group B. Datasets 2, 8, 10 and 15 (in bold/italic) provided A95 values that fall out of the A95min/A95max, range, and hence were not considered for the paleomagnetic data processing and the subsequent Model G curve fitting for the IHMP (see section “IHMP: selection criteria for the paleomagnetic database”).

### IHMP paleomagnetic data processing

From the paleomagnetic database, all VGP angular dispersion data were calculated by means of Eq. (1). Upper and lower limits for *S* (*S*_*u*_ and *S*_*l*_, respectively) were estimated as suggested by the bootstrap method. Obtaining angular dispersion data due to the PSV (*S*_*B*_) can be done by minimization of sampling and measurement errors^25^ by means of the following relationship:

3 $${S}_{B}=\sqrt{{S}^{2}-\frac{{{S}_{W}}^{2}}{\bar{n}}}$$

where $\bar{n}\,\,$and *S*_*w*_ are, respectively, the average number of samples per site and the within-site dispersion. The relation ${S}_{w}^{2}/\bar{n}$ is the correction factor for the within-site dispersion of a given VGP dataset, which is given by^42^:

4 $$\frac{{{S}_{W}}^{2}}{\bar{n}}=0.335{\bar{\alpha }}_{95}^{2}\frac{2{(1+3{\sin }^{2}\lambda )}^{2}}{(5+3{\sin }^{2}\lambda )}$$

where ${\bar{\alpha }}_{95}$ is the mean value of *α*_95_ for the VGP dataset. *S*_*B*_ data are also displayed in Table 1. The mean difference between *S* and *S*_*B*_ is quite small (~1.9°), which could be an indirect indicator for the adequacy of the selection criteria adopted in this work. For the evaluation of VGP dispersion data regarding the paleolatitude for the IHMP, we considered the *S*_*B*_ (λ) data.

#### Model G curve fitting

For evaluation of the paleolatitudinal dependence of the VGP dispersion data to the selected *S*_*B*_ (λ) dataset for the IHMP, we performed a curve fitting based on the Model G (ref. ^31^) by means on the Levenberg–Marquardt method, which is an iterative regression method for solving nonlinear least square problems, by means of a stabilization parameter that assures the convergence of the goal function for a minimum value by choosing Steepest Descent or Gauss-Newton methods (ref. ^45^). It was done by means of the modulus “scipy.optimize.leastsq”, available at the Python online repository *ScyPy* (https://scipy.github.io/devdocs/generated/scipy.optimize.least_squares.html). From the best Model G fitted curve, we carried out the shape parameters *a* and *b* for the *S*_*B*_ (λ) dataset to the IHMP, which will be discussed later.

## Results

### Evaluation of the paleolatitudinal dependence of the VGP dispersion data for the IHMP

**Figure 1:**
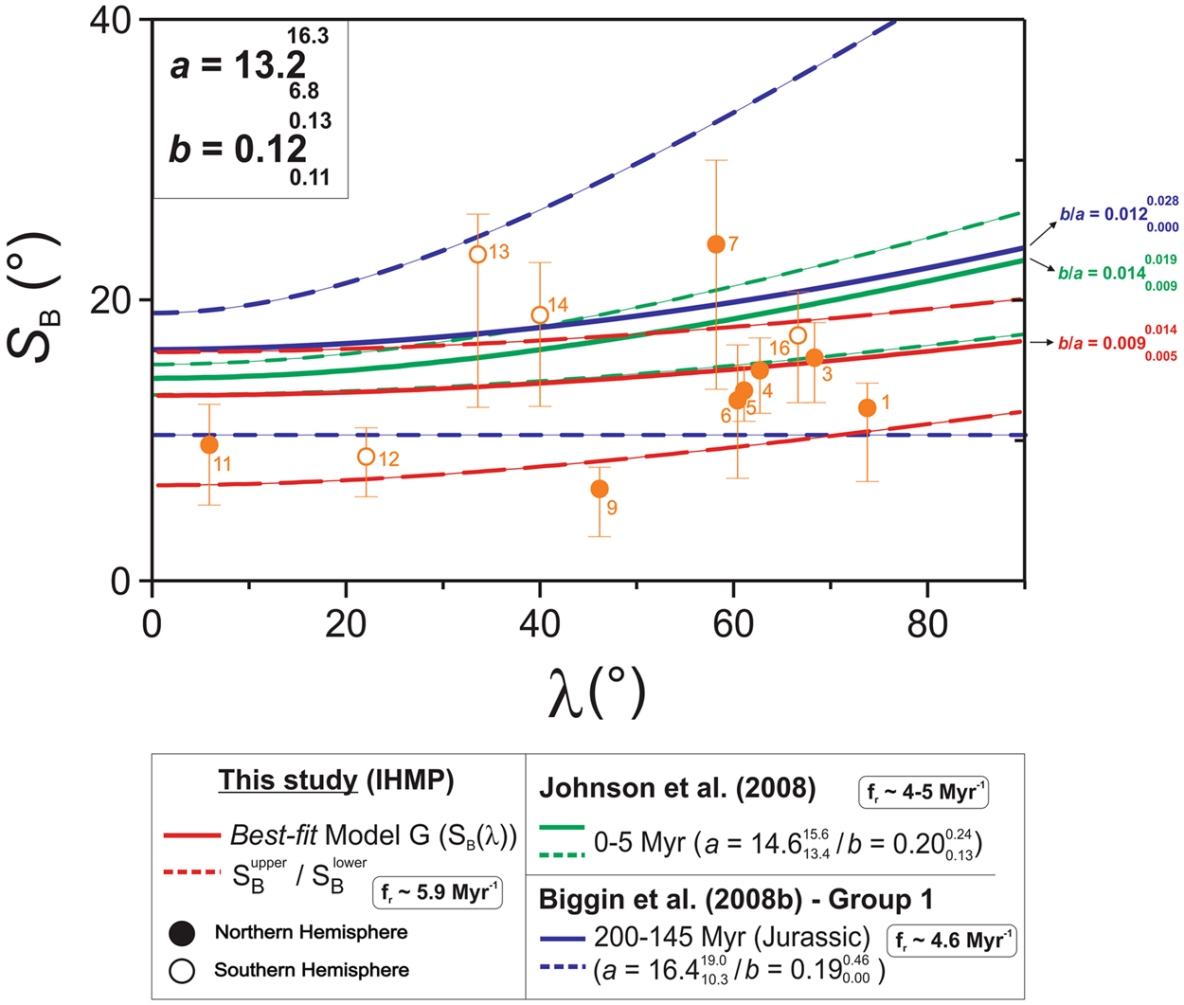
VGP dispersion only due to the PSV (*S*_B_) as a function of paleolatitude in hemispheric projection for the selected paleomagnetic database. Demonstrated together with the IHMP data is the best-fit Model G (ref. ^31^) (red line), associated to its 95% confidence limits (dashed lines). This curve is compared to the *S*(λ) curves for the last 5 Ma (green lines; ref. ^28^) and for the Group 1 dataset (blue) for Jurassic times provided by Biggin *et al*. (ref. ^25^). For each curve the correspondent *b*/*a* ratio is indicated on the right.

By the hemispheric representation of the selected database along with its corresponding paleolatitudes (Fig. 1), it was not possible to observe any evidence for an equatorial asymmetry between the *S*_*B*_ dispersion datasets related to both Southern and Northern hemispheres (open and full circles, respectively) – which could be reasonably explained by the assumption of the GAD hypothesis, as previously discussed by Biggin *et al*. (ref. ^25^). The *S*_B_ (*λ*) distribution, in association to its best fitted Model G curve (which resulted in shape parameters ${13.2}_{6.8}^{16.3}$ and ${0.12}_{0.11}^{0.13}$), clearly exhibits a low paleolatitudinal dependence trending pattern (ranging from *S*_*B*_ ~13.8° to ~17.0° at (paleo)latitudes = 0° and 90°, respectively). All the three *S*_*B*_ (λ) curves exhibit similar shapes, which is compatible to a low (paleo)latitudinal dependence due to smaller antisymmetric contribution during high reversal rate intervals. Nevertheless, the IHMP interval (average reversal rate of ~5.9 Myr^−1^) exhibits higher *S*_*B*_ values at low paleolatitudes in comparison to those reported for lower reversal frequency intervals, as the CNS^25^ (~8.7°) and the PCRS^26^ (~9.4°) – and similar to the observed to the 0–5 Myr and Jurassic intervals, of similar reversal frequency (4–5 Myr^−1^ and 4.6 Myr^−1^, respectively).

Furthermore, in order to compare the observed paleolatitudinal trending pattern and shape of the VGP dispersion curve for the IHMP with other high mean reversal frequency intervals we also demonstrated in Fig. 1, the Model G best-fit curves respectively provided for Jurassic times from Group 1 of Biggin *et al*. (ref. ^25^) and for the last 5 Myr^28^.

All curves exhibit the same low paleolatitudinal trending patterns, which has been discussed in literature (e.g., ref. ^25^ and ref. ^27^) as being due to a major symmetric family contribution in comparison to the influence from the antisymmetric family. Such effect leads to higher (lower) values for the shape parameter *a* (*b*) in comparison to low reversal frequency intervals, as the CNS (Johnson & McFadden, ref. ^4^). The IHMP (red) and Jurassic (blue) curves evolved similarly within the 0–90° paleolatitudinal interval, although the IHMP *S*_*B*_ (λ) curve exhibit lower *S*_*B*_ at lower and higher paleolatitudes. The VGP dispersion curves for both Jurassic and 0–5 Myr intervals provided shape parameters that are compatible to those found for IHMP (Jurassic: $a={16.4}_{10.3}^{19.0}$ and $b={0.19}_{0.00}^{0.46}$; 0–5 Myr: $a={14.6}_{13.4}^{15.6}$ and $b={0.20}_{0.13}^{0.24}$).

It can be noticed that the *b*/*a* ratios – which can be considered as an empirical measure of the relative contribution of the antisymmetric/symmetric harmonic terms^24^ – for the Jurassic ($${0.012}_{0.000}^{0.028}\,)$$ and 0–5 Myr ($${0.014}_{0.009}^{0.019})\,\,$$intervals are slightly higher than the *b*/*a* ratio found for the IHMP ($$={0.009}_{0.005}^{0.014})$$. Additionally, the mean reversal rate for the Jurassic^25^ (~4.6 Myr^−1^) and the 0–5 Myr^28^ (~4–5 Myr^−1^) intervals are quite similar. We estimated the mean average reversal frequency for the IHMP (for more detail, see description in “Evaluation of the time evolution of the *b/a* ratio” section) as ~5.9 Myr^−1^, for the ~266.7–228.7 Myr suggested for this period, which is higher than the previous two intervals. By comparison, the higher (lower) values of mean average reversal frequency (*b/a* ratio) found for IHMP in comparison to the last 5 Myr and Jurassic could indicate the inverse relationship between mean reversal rate and *b/a* ratio, as expected, and the even lower influence of the antisymmetric family for the IHMP.

### Evaluation of the time evolution of the *b*/*a* ratio

As discussed by several authors^13,14,18,20^, the timescale of the anharmonic variations verified along the GPTS are evocative of the mantle convection timescales – which is itself comparable to the variations of the heat flux patterns over the CMB, as suggested by numerical modeling works of mantle convection^46,47^.

**Figure 2:**
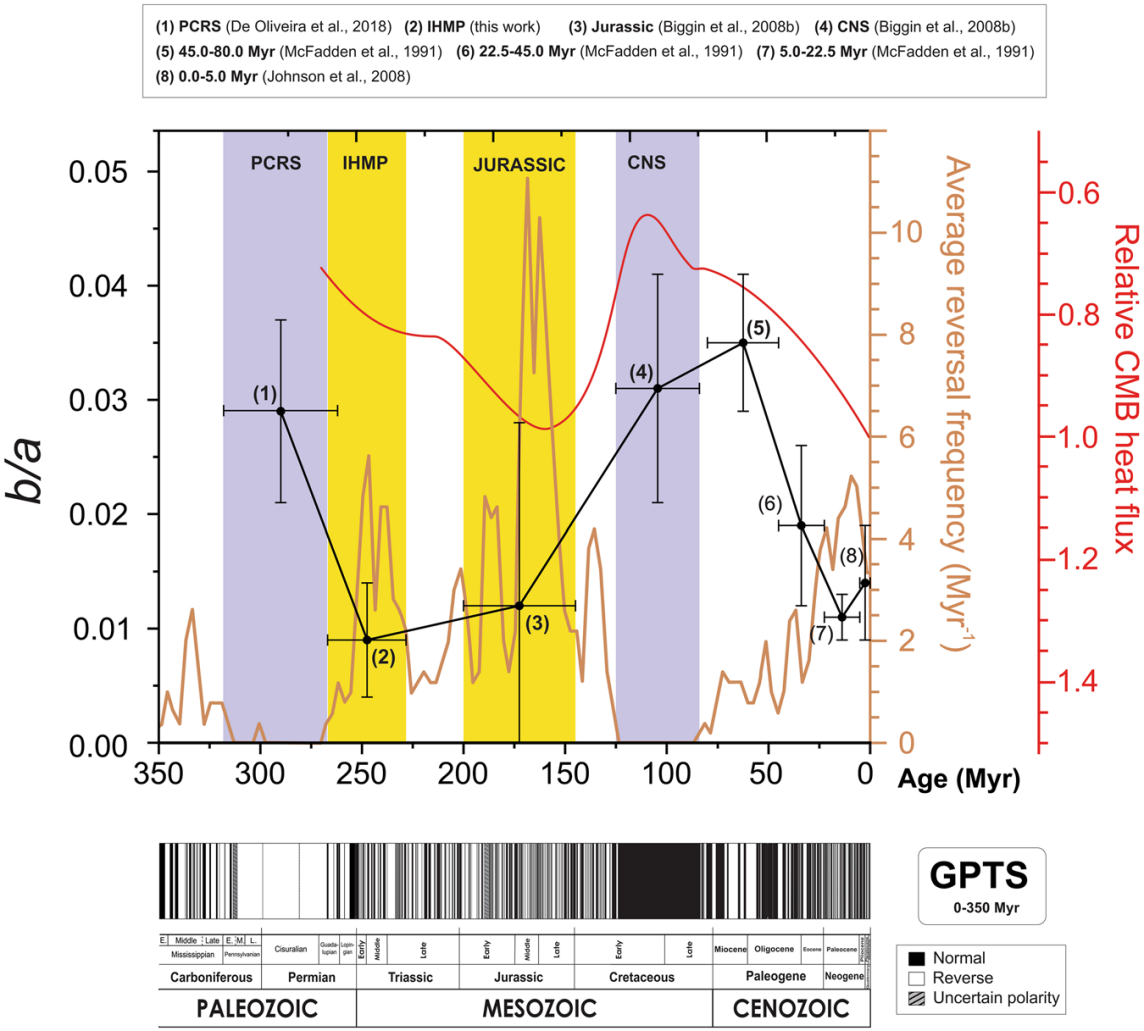
Time evolution for the last 270 Myr between the *b*/*a* ratio (dark circles) (based on calculations provided by different studies – including the IHMP data, provided by this work) and the CMB heat flux variations relative to the present day according to the Olson & Amit (2015) model (smoothed curve in red). Estimates for the average reversal frequency (for the last 350 Myr) are also available for comparison (brown curve).

In order to contribute to this debate, we also conducted an evaluation aiming to track the time evolution of the relative contribution of dipole/non-dipole fields derived from paleomagnetic data – by means of *b*/*a* ratios – and its possible correspondence with time variations in relative CMB heat flux throughout most of the Phanerozoic. The results for *b*/*a* ratios were provided both by this work and other studies, which together comprise contiguous, million-year scale intervals that exhibited high and low mean reversal rates throughout the Phanerozoic: (1) PCRS^26^; (2) IHMP (this study); (3) Jurassic^25^; (4) CNS^25^; (5) 45–80 Myr^27^; (6) 22.5–45.0 Myr^27^; (7) 5.0–22.5 Myr^27^; (8) 0–5 Myr^28^ (Table 2 and Fig. 2). It is important to highlight that, as discussed by Biggin *et al*. (ref. ^25^) the data provided by McFadden *et al*. (ref. ^27^) probably reflect a latitudinal dependence to the VGP scatter by application of a constant within-site error correction in pole-space. Estimates of the time evolution of the relative CMB heat flux for the past 270 Myr, based on temporal variations in relative geomagnetic reversal frequency, followed the recent model proposed by Olson & Amit (ref. ^9^). Their approach is supported by indications from convection-driven numerical dynamos^16,47^ of which the likelihood of the geomagnetic polarity reversals occur is proportion to the increasing of the CMB heat flux on the outer boundary. Additionally, we estimated the average reversal frequency based on the GPTS provided by Gradstein *et al*. (ref. ^48^), by application of a 3 Myr running window in steps of 2 Myr for the past 350 Myr.

**Table 2:** Secondary (symmetric) (${a}_{l}^{u}$) and primary (antisymmetric) (${b}_{l}^{u}$) harmonic terms, and estimates for its relative contribution (*b*/*a* ratio), carried out from Model G fitting curves from this work and other studies based on VGP dispersion analyses for most of the Phanerozoic.

#	Work	Interval (Myr)	$\,{a}_{l}^{u}$	$\,{b}_{l}^{u}$	*b*/*a*
1	De Oliveira *et al*.^26^^(a)^	262.0–318.0	${9.4}_{7.5}^{10.9}$	${0.27}_{0.22}^{0.29}$	${0.029}_{0.021}^{0.037}$
2	**This work**^(b)^	**228**.**7–266**.**7**	${\bf{13}}{\boldsymbol{.}}{{\bf{2}}}_{{\bf{6.8}}}^{{\bf{16.3}}}$	${\bf{0.1}}{{\bf{2}}}_{{\bf{0.11}}}^{{\bf{0.13}}}$	${\bf{0.00}}{{\bf{9}}}_{{\bf{0.004}}}^{{\bf{0.014}}}$
3	Biggin *et al*.^25^^(c)^	145.0–200.0	${16.4}_{10.3}^{19.0}$	${0.19}_{0.00}^{0.46}$	${0.012}_{0.000}^{0.028}$
4	Biggin *et al*.^25^^(d)^	84.0–125.0	${8.7}_{6.3}^{10.7}$	${0.27}_{0.22}^{0.31}$	${0.031}_{0.021}^{0.041}$
5	McFadden *et al*.^27^	45.0–80.0	${9.7}_{8.2}^{11.2}$	${0.34}_{0.31}^{0.37}$	$\,{0.035}_{0.029}^{0.041}$
6	McFadden *et al*.^27^	22.5–45.0	${15.4}_{12.7}^{20.3}$	${0.29}_{0.21}^{0.35}$	$\,{0.019}_{0.012}^{0.026}$
7	McFadden *et al*.^27^	5.0–22.5	${17.8}_{16.9}^{18.7}$	${0.19}_{0.16}^{0.22}$	$\,{0.011}_{0.009}^{0.012}$
8	Johnson *et al*.^28^	0–5.0	${14.6}_{13.4}^{15.6}$	${0.20}_{0.13}^{0.24}$	$\,{0.014}_{0.009}^{0.019}$

u (l): upper (lower) limits for the shape parameters *a* and *b*; ^(a)^PCRS – Permian-Carboniferous Reversed Superchron; ^(b)^IHMP – Illawarra Hyperzone of Mixed Polarity; ^(c)^Jurassic; ^(d)^CNS – Cretaceous Normal Superchron.

It is noticeable that the time evolution of the *b*/*a* ratio matches, in an inverse relationship, the smooth trending pattern for the relative CMB heat flux from the PCRS to the present times, as provided by Olson & Amit (ref. ^9^). It is important to highlight that the *b*/*a* ratios were carried out with PSV analyses from Model G fittings of VGP dispersion curves, which are not of straightforward interpretation in terms of physical processes, because their origins rely on a number of different factors^19^.

Nevertheless, our results point out that the relative contribution of equatorially antisymmetric to symmetric spherical harmonics terms, given by the Model G, could be inversely related to the CMB heat flux variations, indicating that higher axial (non-axial) dipole contributions may be expected for lower (higher) relative CMB heat flux intervals for the last 270 Myr. As discussed previously, high/low *b/a* ratios would be considered, for a given time interval, as a predictor of low/high reversal frequency states^24^ – which in turn could reflects high/low CMB heat flow conditions, as discussed by some authors^47,49,50^.

Such observations would shed some light on the physical meaning of the Model G shape parameters *a* and *b*, what can partially explain the adequacy of this phenomenological model for most of the Phanerozoic. Surely new investigations aiming to extend back in time the *b*/*a* ratio coverage herein presented, and with more time resolution, are demanded to verify the hypothesis.

## Electronic supplementary material

Supplementary Table S1

Supplementary Table S2
