# Author Correction: Paleomagnetic Evidence for Inverse Correspondence between the Relative Contribution of the Axial Dipole Field and CMB Heat Flux for the Past 270 Myr

**DOI:** 10.1038/s41598-020-57827-9

**Published:** 2020-01-15

**Authors:** Daniel Ribeiro Franco, Wellington Paulo de Oliveira, Felipe Barbosa Venâncio de Freitas, Diego Takahashi, Cosme Ferreira da Ponte Neto, Ian Muzy Camarão Peixoto

**Affiliations:** 1 Coordenação de Geofísica, Observatório Nacional, R. Gal. José Cristino, 77, 20921-400, Rio de Janeiro, RJ Brazil; 2 Instituto de Geociências, Universidade Federal Fluminense, Av. Milton Tavares de Souza, S/N, 24210-346 Niterói, RJ Brazil

Correction to: *Scientific Reports* 10.1038/s41598-018-36494-x, published online 22 January 2019

In Figure 2 there is an error in the labelling of the geological timescale. The correct figure appears below as Fig. 1.

**Figure 1:**
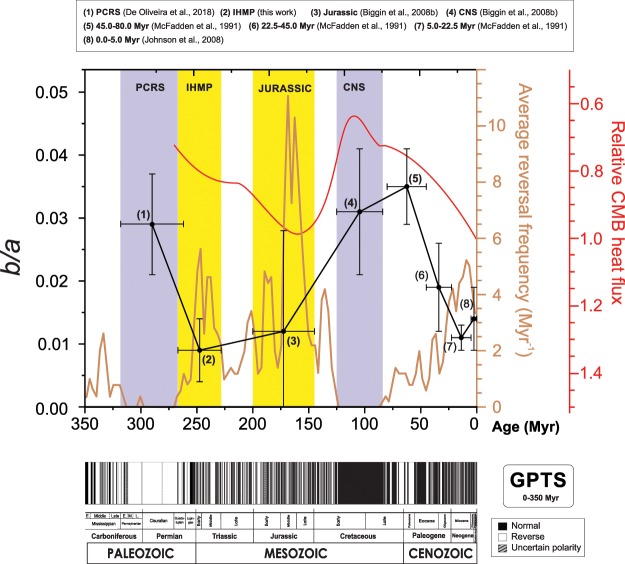
.

