# Supplementary material for: Paleomagnetic Evidence for Inverse Correspondence between the Relative Contribution of the Axial Dipole Field and CMB Heat Flux for the Past 270 Myr
Source: Sci Rep. 2019 Jan 22;9:282. doi: 10.1038/s41598-018-36494-x (PMC6342988; doi:10.1038/s41598-018-36494-x)
Supplement: Supplementary file 1 — Supplementary Table S1 [file 41598_2018_36494_MOESM1_ESM.docx]

Supplementary Information

**Paleomagnetic evidence for inverse correspondence between the relative contribution of the axial dipole field and CMB heat flux for the past 270 Myr**

Daniel Ribeiro Franco^1*^, Wellington Paulo de Oliveira^1^, Felipe Barbosa Venâncio de Freitas^1^, Diego Takahashi^1^, Cosme Ferreira da Ponte Neto^1^, Ian Muzy Camarão Peixoto^2^.

^1^Coordenação de Geofísica, Observatório Nacional, R. Gal. José Cristino, 77, 20921-400 Rio de Janeiro, RJ, Brazil,

^2^Instituto de Geociências, Universidade Federal Fluminense, Av. Milton Tavares de Souza, S/N, 24210-346 Niterói, RJ, Brazil

*To whom correspondence should be addressed. E-mail: drfranco@on.br

| \| **Supplementary Table 1:** Directional and statistical data (site latitude (λ_S_) and longitude (ϕ_S_); declination (D_ChRM_) and inclination (I_ChRM_) of characteristic remanent magnetization; α_95_: 95% confidence cone) from the selected paleomagnetic database (according to the selection criteria discussed at section 2.1). Numbering of the datasets (*) are in accordance to Table 1. References are listed in the Supplementary Table S2. \| \| \| \| \| \| \| \| \| \| \| \| \| \| \| \| \| \| \| --- \| --- \| --- \| --- \| --- \| --- \| --- \| --- \| --- \| --- \| --- \| --- \| --- \| --- \| --- \| --- \| --- \| --- \| \| **(1) Kravchinsky et al. (2002) *** \| \| \| \| \| \| **(2) Veselovskiy et al. (2012) *** \| \| \| \| \| \| **(3) Latyshev et al. (2018) *** \| \| \| \| \| \| \| **Site** \| **λ_s_ (°N)** \| **ϕ_s_ (°E)** \| **D_ChRM_ (°)** \| **I_ChRM_ (°)** \| **α_95_  (°)** \| **Site** \| **λ_s_ (°N)** \| **ϕ_s_ (°E)** \| **D_ChRM_ (°)** \| **I_ChRM_ (°)** \| **α_95_  (°)** \| **Site** \| **λ_s_ (°N)** \| **ϕ_s_ (°E)** \| **D_ChRM_ (°)** \| **I_ChRM_ (°)** \| **α_95_  (°)** \| \| Aikh-1 \| 66.17 \| 111.33 \| 43.5 \| 81.5 \| 13.7 \| Outcrop 26 \| 71.6 \| 114.5 \| 31.0 \| 85.0 \| 8.9 \| 1–14 \| 63.83 \| 97.94 \| 123.1 \| 80.9 \| 2.6 \| \| Aikh-2 \| 66.17 \| 111.33 \| 22.0 \| 89.2 \| 10.8 \| Outcrop 58 \| 71.6 \| 114.5 \| 59.0 \| 69.0 \| 23.0 \| 2–14 \| 63.74 \| 97.99 \| 66.1 \| 77.6 \| 5.8 \| \| Aikh-3 \| 66.17 \| 111.33 \| 116.0 \| 72.8 \| 12.9 \| Outcrop 72 \| 71.6 \| 114.5 \| 63.0 \| 79.0 \| 11.0 \| 3–14 \| 63.78 \| 97.41 \| 166.2 \| 82.6 \| 2.5 \| \| Aikh-4 \| 66.17 \| 111.33 \| 30.4 \| 87.2 \| 3.6 \| Outcrop 74 \| 71.6 \| 114.5 \| 105.0 \| 79.0 \| 10.2 \| 4–14 \| 63.80 \| 96.65 \| 130.3 \| 79.3 \| 2.1 \| \| Aikh-5 \| 66.17 \| 111.33 \| 85.0 \| 76.4 \| 2.6 \| Outcrop 34 \| 71.6 \| 114.5 \| 61.0 \| 82.0 \| 7.2 \| 9–14 \| 64.19 \| 94.60 \| 71.3 \| 81.6 \| 1.9 \| \| Aikh-6 \| 66.17 \| 111.33 \| 155.0 \| 83.7 \| 14.9 \| Outcrop 58' \| 71.6 \| 114.5 \| 179.0 \| 75.0 \| 12.8 \| 10–14 \| 64.18 \| 94.35 \| 95.6 \| 81.3 \| 1.8 \| \| Aikh-7 \| 66.17 \| 111.33 \| 134.5 \| 79.6 \| 7.7 \| dike 28 \| 71.6 \| 114.5 \| 82.0 \| 73.0 \| 7.0 \| 11–14 \| 64.18 \| 93.84 \| 109.9 \| 67.7 \| 2.9 \| \| Aikh-8 \| 66.17 \| 111.33 \| 129.9 \| 71.7 \| 13.1 \| dike 30 \| 71.6 \| 114.5 \| 176.0 \| 64.0 \| 5.4 \| 13–14 \| 64.36 \| 93.24 \| 95.2 \| 70.7 \| 4.6 \| \| Aikh-9 \| 66.17 \| 111.33 \| 98.4 \| 86.4 \| 3.5 \| dike 56 \| 71.6 \| 114.5 \| 67.0 \| 79.0 \| 33.5 \| 14–14 \| 64.43 \| 92.93 \| 80.8 \| 72.7 \| 3.0 \| \| Aikh-10 \| 66.17 \| 111.33 \| 78.2 \| 72.6 \| 8.6 \| Outcrop 57 \| 71.6 \| 114.5 \| 179.0 \| 71.0 \| 12.9 \| 15–14 \| 64.38 \| 92.64 \| 133.0 \| 77.0 \| 2.4 \| \|  \|  \|  \| **mean α_95_  (°):** \| \| 9.1 \| Outcrop 71 \| 71.6 \| 114.5 \| 151.0 \| 64.0 \| 48.1 \| 18–14 \| 64.43 \| 91.87 \| 93.7 \| 81.7 \| 3.3 \| \|  \|  \|  \|  \|  \|  \| dike 76 \| 71.6 \| 114.5 \| 131.0 \| 85.0 \| 5.4 \| 21–14 \| 64.59 \| 90.93 \| 142.6 \| 74.7 \| 2.0 \| \|  \|  \|  \|  \|  \|  \| paleovolcano 79 \| 71.6 \| 114.5 \| 121.0 \| -70.0 \| 15.6 \| 22–14 \| 64.74 \| 90.73 \| 89.0 \| 78.5 \| 6.4 \| \|  \|  \|  \|  \|  \|  \| dike 4 \| 70.7 \| 117.2 \| 89.0 \| 69.0 \| 14.1 \| 25–14 \| 65.03 \| 90.09 \| 82.6 \| 81.6 \| 4.7 \| \|  \|  \|  \|  \|  \|  \| dike 6 \| 70.7 \| 117.2 \| 79.0 \| 77.0 \| 18.2 \| 26–14 \| 65.08 \| 90.99 \| 29.4 \| 76.1 \| 6.5 \| \|  \|  \|  \|  \|  \|  \| dike 8a \| 70.7 \| 117.2 \| 123.0 \| 74.0 \| 13.1 \| 27–14 \| 65.16 \| 89.99 \| 50.0 \| 70.6 \| 8.9 \| \|  \|  \|  \|  \|  \|  \| dike 8b \| 70.7 \| 117.2 \| 90.0 \| 72.0 \| 6.9 \| 28–14 \| 65.27 \| 89.26 \| 112.0 \| 83.0 \| 6.8 \| \|  \|  \|  \|  \|  \|  \| sill 17 \| 70.7 \| 117.2 \| 106.0 \| 72.0 \| 5.2 \| 29–14 \| 65.46 \| 89.97 \| 141.5 \| 71.6 \| 2.4 \| \|  \|  \|  \|  \|  \|  \|  \|  \|  \| **mean α_95_  (°):** \| \| 14.3 \| 31-14 \| 65.60 \| 90.07 \| 69.5 \| 75.2 \| 7.2 \| \|  \|  \|  \|  \|  \|  \|  \|  \|  \|  \|  \|  \| 32–14 \| 65.69 \| 89.81 \| 105.9 \| 63.6 \| 5.6 \| \|  \|  \|  \|  \|  \|  \|  \|  \|  \|  \|  \|  \| 34–14 \| 65.85 \| 89.26 \| 70.7 \| 75.8 \| 3.4 \| \|  \|  \|  \|  \|  \|  \|  \|  \|  \|  \|  \|  \| 15–12 \| 64.11 \| 98.09 \| 39.1 \| 83.4 \| 14.2 \| \|  \|  \|  \|  \|  \|  \|  \|  \|  \|  \|  \|  \| 11–12 \| 64.27 \| 100.24 \| 42.6 \| 78.6 \| 10.4 \| \|  \|  \|  \|  \|  \|  \|  \|  \|  \|  \|  \|  \| 5–14 \| 63.89 \| 95.91 \| 300.8 \| -77.0 \| 3.4 \| \|  \|  \|  \|  \|  \|  \|  \|  \|  \|  \|  \|  \| 6–14 \| 63.93 \| 95.79 \| 301.5 \| -71.9 \| 3.2 \| \|  \|  \|  \|  \|  \|  \|  \|  \|  \|  \|  \|  \| 7–14 \| 64.02 \| 95.36 \| 259.4 \| -74.9 \| 2.1 \| \|  \|  \|  \|  \|  \|  \|  \|  \|  \|  \|  \|  \| 12–14 \| 64.25 \| 93.55 \| 216.0 \| -66.8 \| 2.8 \| \|  \|  \|  \|  \|  \|  \|  \|  \|  \|  \|  \|  \| 16–14 \| 64.36 \| 92.32 \| 322.4 \| -65.8 \| 2.4 \| \|  \|  \|  \|  \|  \|  \|  \|  \|  \|  \|  \|  \| 17–14 \| 64.34 \| 92.16 \| 285.2 \| -76.0 \| 2.6 \| \|  \|  \|  \|  \|  \|  \|  \|  \|  \|  \|  \|  \| 19–14 \| 64.46 \| 91.40 \| 284.8 \| -77.8 \| 4.6 \| \|  \|  \|  \|  \|  \|  \|  \|  \|  \|  \|  \|  \| 20–14 \| 64.52 \| 91.17 \| 300.9 \| -80.6 \| 4.1 \| \|  \|  \|  \|  \|  \|  \|  \|  \|  \|  \|  \|  \| 23–14 \| 64.77 \| 90.61 \| 261.2 \| -84.2 \| 5.1 \| \|  \|  \|  \|  \|  \|  \|  \|  \|  \|  \|  \|  \| 24–14 \| 64.91 \| 90.30 \| 273.0 \| -79.7 \| 2.0 \| \|  \|  \|  \|  \|  \|  \|  \|  \|  \|  \|  \|  \| 30–14 \| 65.54 \| 90.00 \| 260.9 \| -80.0 \| 2.8 \| \|  \|  \|  \|  \|  \|  \|  \|  \|  \|  \|  \|  \| 33–14 \| 65.76 \| 89.52 \| 210.7 \| -76.1 \| 7.1 \| \|  \|  \|  \|  \|  \|  \|  \|  \|  \|  \|  \|  \|  \|  \|  \| **mean α_95_  (°):** \| \| 4.4 \| |
| --- | --- | --- | --- | --- | --- | --- | --- | --- | --- | --- | --- | --- | --- | --- | --- | --- | --- | --- | --- | --- | --- | --- | --- | --- | --- | --- | --- | --- | --- | --- | --- | --- | --- | --- | --- | --- | --- | --- | --- | --- | --- | --- | --- | --- | --- | --- | --- | --- | --- | --- | --- | --- | --- | --- | --- | --- | --- | --- | --- | --- | --- | --- | --- | --- | --- | --- | --- | --- | --- | --- | --- | --- | --- | --- | --- | --- | --- | --- | --- | --- | --- | --- | --- | --- | --- | --- | --- | --- | --- | --- | --- | --- | --- | --- | --- | --- | --- | --- | --- | --- | --- | --- | --- | --- | --- | --- | --- | --- | --- | --- | --- | --- | --- | --- | --- | --- | --- | --- | --- | --- | --- | --- | --- | --- | --- | --- | --- | --- | --- | --- | --- | --- | --- | --- | --- | --- | --- | --- | --- | --- | --- | --- | --- | --- | --- | --- | --- | --- | --- | --- | --- | --- | --- | --- | --- | --- | --- | --- | --- | --- | --- | --- | --- | --- | --- | --- | --- | --- | --- | --- | --- | --- | --- | --- | --- | --- | --- | --- | --- | --- | --- | --- | --- | --- | --- | --- | --- | --- | --- | --- | --- | --- | --- | --- | --- | --- | --- | --- | --- | --- | --- | --- | --- | --- | --- | --- | --- | --- | --- | --- | --- | --- | --- | --- | --- | --- | --- | --- | --- | --- | --- | --- | --- | --- | --- | --- | --- | --- | --- | --- | --- | --- | --- | --- | --- | --- | --- | --- | --- | --- | --- | --- | --- | --- | --- | --- | --- | --- | --- | --- | --- | --- | --- | --- | --- | --- | --- | --- | --- | --- | --- | --- | --- | --- | --- | --- | --- | --- | --- | --- | --- | --- | --- | --- | --- | --- | --- | --- | --- | --- | --- | --- | --- | --- | --- | --- | --- | --- | --- | --- | --- | --- | --- | --- | --- | --- | --- | --- | --- | --- | --- | --- | --- | --- | --- | --- | --- | --- | --- | --- | --- | --- | --- | --- | --- | --- | --- | --- | --- | --- | --- | --- | --- | --- | --- | --- | --- | --- | --- | --- | --- | --- | --- | --- | --- | --- | --- | --- | --- | --- | --- | --- | --- | --- | --- | --- | --- | --- | --- | --- | --- | --- | --- | --- | --- | --- | --- | --- | --- | --- | --- | --- | --- | --- | --- | --- | --- | --- | --- | --- | --- | --- | --- | --- | --- | --- | --- | --- | --- | --- | --- | --- | --- | --- | --- | --- | --- | --- | --- | --- | --- | --- | --- | --- | --- | --- | --- | --- | --- | --- | --- | --- | --- | --- | --- | --- | --- | --- | --- | --- | --- | --- | --- | --- | --- | --- | --- | --- | --- | --- | --- | --- | --- | --- | --- | --- | --- | --- | --- | --- | --- | --- | --- | --- | --- | --- | --- | --- | --- | --- | --- | --- | --- | --- | --- | --- | --- | --- | --- | --- | --- | --- | --- | --- | --- | --- | --- | --- | --- | --- | --- | --- | --- | --- | --- | --- | --- | --- | --- | --- | --- | --- | --- | --- | --- | --- | --- | --- | --- | --- | --- | --- | --- | --- | --- | --- | --- | --- | --- | --- | --- | --- | --- | --- | --- | --- | --- | --- | --- | --- | --- | --- | --- | --- | --- | --- | --- | --- | --- | --- | --- | --- | --- | --- | --- | --- | --- | --- | --- | --- | --- | --- | --- | --- | --- | --- | --- | --- | --- | --- | --- | --- | --- | --- | --- | --- | --- | --- | --- | --- | --- | --- | --- | --- | --- | --- | --- | --- | --- | --- | --- | --- | --- | --- | --- | --- | --- | --- | --- | --- | --- | --- | --- | --- | --- | --- | --- | --- | --- | --- | --- | --- | --- | --- | --- | --- | --- | --- | --- | --- | --- | --- | --- | --- | --- | --- | --- | --- | --- | --- | --- | --- | --- | --- | --- | --- | --- | --- | --- | --- | --- | --- | --- | --- | --- | --- | --- | --- | --- | --- | --- | --- | --- | --- | --- | --- | --- | --- | --- | --- | --- | --- | --- | --- | --- | --- | --- | --- | --- | --- | --- | --- | --- | --- | --- | --- | --- | --- | --- | --- | --- | --- | --- | --- | --- | --- | --- | --- | --- | --- | --- | --- | --- | --- | --- | --- | --- | --- | --- | --- | --- | --- | --- | --- | --- | --- | --- | --- | --- | --- | --- | --- | --- | --- | --- | --- | --- | --- | --- | --- | --- | --- | --- | --- | --- | --- | --- | --- | --- | --- | --- | --- | --- | --- | --- | --- | --- | --- | --- | --- | --- | --- |

| \| **Supplementary Table 1 (continuation):** Directional and statistical data (site latitude (λ_S_) and longitude (ϕ_S_); declination (D_ChRM_) and inclination (I_ChRM_) of characteristic remanent magnetization; α_95_: 95% confidence cone) from the selected paleomagnetic database (according to the selection criteria discussed at section 2.1). Numbering of the datasets (*) are in accordance to Table 1. References are listed in "References (Table 1)" worksheet. \| \| \| \| \| \| \| \| \| \| \| \| \| \| \| \| \| \| \| \| \| \| \| \| \| --- \| --- \| --- \| --- \| --- \| --- \| --- \| --- \| --- \| --- \| --- \| --- \| --- \| --- \| --- \| --- \| --- \| --- \| --- \| --- \| --- \| --- \| --- \| --- \| \| **(4) Heunemann et al. (2004) *** \| \| \| \| \| \| **(5) Pavlov et al. (2011) *** \| \| \| \| \| \| \| **(6) Veselovskiy et al. (2012) *** \| \| \| \| \| \| \| \| \| \| \| \| **Site** \| **λ_s_ (°N)** \| **ϕ_s_ (°E)** \| **D_ChRM_ (°)** \| **I_ChRM_ (°)** \| **α_95_  (°)** \| **Site** \| **λ_s_ (°N)** \| **ϕ_s_ (°E)** \| **D_ChRM_ (°)** \| \| **I_ChRM_ (°)** \| **α_95_  (°)** \| **Site** \| **λ_s_ (°N)** \| \| **ϕ_s_ (°E)** \| \| **D_ChRM_ (°)** \| \| **I_ChRM_ (°)** \| \| **α_95_  (°)** \| \| \| KMX4 \| 70.37 \| 90.01 \| 37.0 \| 77.0 \| 5.0 \| hard 1 \| 71.3 \| 102.5 \| 101.3 \| \| 65.3 \| 16.1 \| 17-1 \| 70.9 \| \| 100.6 \| \| 101.8 \| \| 68.8 \| \| 15.4 \| \| \| KMX3 \| 70.37 \| 90.01 \| 47.0 \| 74.0 \| 5.6 \| hard 2 \| 71.3 \| 102.5 \| 128.5 \| \| 67.4 \| 9.3 \| 17-2 \| 70.9 \| \| 100.6 \| \| 131.2 \| \| 66.3 \| \| 20.0 \| \| \| KMX2 \| 70.37 \| 90.01 \| 68.0 \| 72.0 \| 2.6 \| hard 3 \| 71.3 \| 102.5 \| 125.6 \| \| 67.4 \| 7.5 \| 17-3 \| 70.9 \| \| 100.6 \| \| 91.9 \| \| 59.6 \| \| 25.2 \| \| \| KMX1 \| 70.37 \| 90.01 \| 75.0 \| 72.0 \| 2.4 \| hard 4 \| 71.3 \| 102.5 \| 131.3 \| \| 71.8 \| 14.2 \| 17-4 \| 70.9 \| \| 100.6 \| \| 51.5 \| \| 70.4 \| \| 5.4 \| \| \| KM3 \| 70.37 \| 90.01 \| 75.0 \| 73.0 \| 2.7 \| hard 5 \| 71.3 \| 102.5 \| 146.0 \| \| 70.1 \| 6.2 \| 17-5 \| 70.9 \| \| 100.6 \| \| 24.3 \| \| 74.5 \| \| 7.6 \| \| \| KM1 \| 70.37 \| 90.01 \| 69.0 \| 72.0 \| 3.7 \| hard 6 \| 71.3 \| 102.5 \| 109.0 \| \| 70.0 \| 24.6 \| 17-6 \| 70.9 \| \| 100.6 \| \| 75.3 \| \| 80.3 \| \| 115.2 \| \| \| HR13 \| 70.37 \| 90.01 \| 59.0 \| 81.0 \| 4.2 \| medv 1 \| 71.3 \| 102.5 \| 134.7 \| \| 71.1 \| 9.0 \| 17-7 \| 70.9 \| \| 100.6 \| \| 106.7 \| \| 79.4 \| \| 75.4 \| \| \| HR11 \| 70.37 \| 90.01 \| 50.0 \| 75.0 \| 4.4 \| medv 2 \| 71.3 \| 102.5 \| 130.6 \| \| 68.9 \| 8.2 \| 17-8 \| 70.9 \| \| 100.6 \| \| 133.6 \| \| 71.0 \| \| 12.2 \| \| \| HR7 \| 70.37 \| 90.01 \| 96.0 \| 73.0 \| 6.6 \| medv 3 \| 71.3 \| 102.5 \| 81.9 \| \| 81.2 \| 8.0 \| 17-9 \| 70.9 \| \| 100.6 \| \| 106.7 \| \| 67.6 \| \| 13.6 \| \| \| HR6 \| 70.37 \| 90.01 \| 109.0 \| 64.0 \| 2.0 \| medv 4 \| 71.3 \| 102.5 \| 68.5 \| \| 86.9 \| 8.3 \| 17-10 \| 70.9 \| \| 100.6 \| \| 46.4 \| \| 77.0 \| \| 11.5 \| \| \| HR5 \| 70.37 \| 90.01 \| 105.0 \| 66.0 \| 5.8 \| flow 0 \| 71.3 \| 102.5 \| 167.6 \| \| 79.5 \| 9.3 \| 17-11 \| 70.9 \| \| 100.6 \| \| 86.9 \| \| 74.1 \| \| 18.2 \| \| \| HR3 \| 70.37 \| 90.01 \| 85.0 \| 53.0 \| 4.0 \| flow 1 \| 71.3 \| 102.5 \| 141.5 \| \| 86.8 \| 5.1 \| 17-12 \| 70.9 \| \| 100.6 \| \| 111.8 \| \| 73.5 \| \| 24.2 \| \| \| HR2 \| 70.37 \| 90.01 \| 94.0 \| 61.0 \| 2.5 \| flow 2 \| 71.3 \| 102.5 \| 149.4 \| \| 82.4 \| 8.2 \| 17-13 \| 70.9 \| \| 100.6 \| \| 87.4 \| \| 85.3 \| \| 4.5 \| \| \| MK13 \| 70.37 \| 90.01 \| 95.0 \| 71.0 \| 2.6 \| flow 3 \| 71.3 \| 102.5 \| 119.6 \| \| 86.7 \| 5.6 \| 17-14 \| 70.9 \| \| 100.6 \| \| 134.4 \| \| 75.3 \| \| 14.7 \| \| \| MK12 \| 70.37 \| 90.01 \| 100.0 \| 70.0 \| 5.5 \| flow 4 \| 71.3 \| 102.5 \| 113.1 \| \| 79.7 \| 4.4 \| 17-15 \| 70.9 \| \| 100.6 \| \| 71.1 \| \| 72.5 \| \| 10.9 \| \| \| MK11 \| 70.37 \| 90.01 \| 90.0 \| 76.0 \| 3.7 \| flow 5 \| 71.3 \| 102.5 \| 120.3 \| \| 75.8 \| 5.0 \| 17-16 \| 70.9 \| \| 100.6 \| \| 71.4 \| \| 74.7 \| \| 16.1 \| \| \| MK10 \| 70.37 \| 90.01 \| 89.0 \| 66.0 \| 6.7 \| flow 6 \| 71.3 \| 102.5 \| 98.2 \| \| 78.6 \| 6.2 \| 17-17 \| 70.9 \| \| 100.6 \| \| 65.1 \| \| 77.2 \| \| 14.5 \| \| \| MK9 \| 70.37 \| 90.01 \| 68.0 \| 67.0 \| 3.4 \| flow 6b \| 71.3 \| 102.5 \| 99.1 \| \| 71.8 \| 4.3 \| 17-18 \| 70.9 \| \| 100.6 \| \| 93.7 \| \| 78.2 \| \| 11.4 \| \| \| MK8 \| 70.37 \| 90.01 \| 106.0 \| 79.0 \| 3.8 \| flow 7 \| 71.3 \| 102.5 \| 178.8 \| \| 82.6 \| 4.9 \| 17-19 \| 70.9 \| \| 100.6 \| \| 108.0 \| \| 53.0 \| \| 24.2 \| \| \| MK7 \| 70.37 \| 90.01 \| 95.0 \| 77.0 \| 4.1 \| flow 8 \| 71.3 \| 102.5 \| 162.8 \| \| 76.0 \| 4.9 \| 17-20 \| 70.9 \| \| 100.6 \| \| 102.0 \| \| 65.5 \| \| 10.4 \| \| \| MK5 \| 70.37 \| 90.01 \| 123.0 \| 82.0 \| 5.0 \| flow 9 \| 71.3 \| 102.5 \| 162.6 \| 78.8 \| \| 4.7 \|  \|  \| \|  \| \| **mean α_95_  (°):** \| \| \| \| 22.5 \| \| \| MK4 \| 70.37 \| 90.01 \| 66.0 \| 74.0 \| 3.1 \| flow 10 \| 71.3 \| 102.5 \| 191.2 \| \| 78.1 \| 4.6 \|  \|  \| \|  \| \|  \| \|  \| \|  \| \| \| MK 3 \| 70.37 \| 90.01 \| 84.0 \| 72.0 \| 2.3 \| flow 13 \| 71.3 \| 102.5 \| 109.8 \| \| 84.3 \| 10.9 \|  \|  \| \|  \| \|  \| \|  \| \|  \| \| \| MK2 \| 70.37 \| 90.01 \| 161.0 \| 75.0 \| 2.8 \| flow 14 \| 71.3 \| 102.5 \| 101.7 \| \| 69.9 \| 10.1 \|  \|  \| \|  \| \|  \| \|  \| \|  \| \| \| MK1 \| 70.37 \| 90.01 \| 141.0 \| 78.0 \| 4.2 \| flow 15 \| 71.3 \| 102.5 \| 103.2 \| \| 70.1 \| 5.5 \|  \|  \| \|  \| \|  \| \|  \| \|  \| \| \| MK0 \| 70.37 \| 90.01 \| 110.0 \| 75.0 \| 2.6 \| flow 16 \| 71.3 \| 102.5 \| 110.6 \| \| 78.0 \| 4.1 \|  \|  \| \|  \| \|  \| \|  \| \|  \| \| \| MR1 \| 70.37 \| 90.01 \| 117.0 \| 77.0 \| 2.3 \| flow 1718 \| 71.3 \| 102.5 \| 211.9 \| \| 83.9 \| 8.8 \|  \|  \| \|  \| \|  \| \|  \| \|  \| \| \| MR2 \| 70.37 \| 90.01 \| 115.0 \| 78.0 \| 3.7 \| flow 19 \| 71.3 \| 102.5 \| 154.5 \| \| 78.8 \| 6.5 \|  \|  \| \|  \| \|  \| \|  \| \|  \| \| \| MR4 \| 70.37 \| 90.01 \| 73.0 \| 76.0 \| 3.6 \| flow 20 \| 71.3 \| 102.5 \| 220.7 \| \| 75.2 \| 5.2 \|  \|  \| \|  \| \|  \| \|  \| \|  \| \| \| MR6 \| 70.37 \| 90.01 \| 96.0 \| 74.0 \| 3.1 \| flow 21 \| 71.3 \| 102.5 \| 156.9 \| \| 79.5 \| 9.4 \|  \|  \| \|  \| \|  \| \|  \| \|  \| \| \| MR7 \| 70.37 \| 90.01 \| 89.0 \| 77.0 \| 2.8 \| flow 22 \| 71.3 \| 102.5 \| 126.0 \| \| 79.5 \| 10.0 \|  \|  \| \|  \| \|  \| \|  \| \|  \| \| \| MR9 \| 70.37 \| 90.01 \| 187.0 \| 82.0 \| 2.9 \| flow 23 \| 71.3 \| 102.5 \| 113.9 \| \| 64.8 \| 9.9 \|  \|  \| \|  \| \|  \| \|  \| \|  \| \| \| MR11 \| 70.37 \| 90.01 \| 136.0 \| 81.0 \| 2.0 \| flow 25 \| 71.3 \| 102.5 \| 156.6 \| \| 72.0 \| 5.6 \|  \|  \| \|  \| \|  \| \|  \| \|  \| \| \| MR20 \| 70.37 \| 90.01 \| 98.0 \| 78.0 \| 2.1 \| flow 26 \| 71.3 \| 102.5 \| 104.2 \| \| 68.1 \| 4.3 \|  \|  \| \|  \| \|  \| \|  \| \|  \| \| \| MR21 \| 70.37 \| 90.01 \| 91.0 \| 80.0 \| 3.7 \| fl 1 \| 71.3 \| 102.5 \| 117.6 \| \| 71.8 \| 4.5 \|  \|  \| \|  \| \|  \| \|  \| \|  \| \| \| MR22 \| 70.37 \| 90.01 \| 77.0 \| 77.0 \| 2.7 \| flow 2 \| 71.3 \| 102.5 \| 114.6 \| \| 74.8 \| 3.6 \|  \|  \| \|  \| \|  \| \|  \| \|  \| \| \| MR23 \| 70.37 \| 90.01 \| 91.0 \| 77.0 \| 5.8 \| flow 3 \| 71.3 \| 102.5 \| 104.5 \| \| 75.3 \| 3.0 \|  \|  \| \|  \| \|  \| \|  \| \|  \| \| \| ND1 \| 70.37 \| 90.01 \| 100.0 \| 73.0 \| 1.5 \| flow 4 \| 71.3 \| 102.5 \| 112.6 \| \| 75.4 \| 5.0 \|  \|  \| \|  \| \|  \| \|  \| \|  \| \| \| ND3 \| 70.37 \| 90.01 \| 40.0 \| 81.0 \| 2.1 \| flow 5 \| 71.3 \| 102.5 \| 105.1 \| \| 62.2 \| 10.7 \|  \|  \| \|  \| \|  \| \|  \| \|  \| \| \| ND4 \| 70.37 \| 90.01 \| 72.0 \| 75.0 \| 4.4 \| flow 12 \| 71.3 \| 102.5 \| 129.1 \| \| 71.2 \| 11.3 \|  \|  \| \|  \| \|  \| \|  \| \|  \| \| \| ND5 \| 70.37 \| 90.01 \| 54.0 \| 72.0 \| 1.8 \| flow 13 \| 71.3 \| 102.5 \| 127.0 \| \| 73.5 \| 9.2 \|  \|  \| \|  \| \|  \| \|  \| \|  \| \| \|  \|  \|  \| **mean α_95_  (°):** \| \| 3.6 \| flow 14 \| 71.3 \| 102.5 \| 90.1 \| \| 60.8 \| 20.8 \|  \|  \| \|  \| \|  \| \|  \| \|  \| \| \|  \|  \|  \|  \|  \|  \| flow 15 \| 71.3 \| 102.5 \| 103.7 \| \| 64.9 \| 7.5 \|  \|  \| \|  \| \|  \| \|  \| \|  \| \| \|  \|  \|  \|  \|  \|  \| **(4) Pavlov et al. (2011) * - continuation** \| \| \| \| \| \| \|  \| \|  \| \|  \| \|  \| \|  \| \|  \| \|  \|  \|  \|  \|  \|  \| \|  \|  \|  \|  \|  \|  \| **Site** \| **λ_s_ (°N)** \| **ϕ_s_ (°E)** \| **D_ChRM_ (°)** \| \| **I_ChRM_ (°)** \| **α_95_  (°)** \|  \|  \| \|  \| \|  \| \|  \| \|  \| \| \|  \|  \|  \|  \|  \|  \| 15crust \| 71.3 \| 102.5 \| 89.8 \| \| 67.3 \| 8.3 \|  \|  \| \|  \| \|  \| \|  \| \|  \| \| \|  \|  \|  \|  \|  \|  \| fl 16 \| 71.3 \| 102.5 \| 96.8 \| \| 63.5 \| 7.6 \|  \|  \| \|  \| \|  \| \|  \| \|  \| \| \|  \|  \|  \|  \|  \|  \| fl 17 \| 71.3 \| 102.5 \| 81.6 \| \| 61.1 \| 6.5 \|  \|  \| \|  \| \|  \| \|  \| \|  \| \| \|  \|  \|  \|  \|  \|  \| fl 18 \| 71.3 \| 102.5 \| 76.4 \| \| 73.4 \| 5.3 \|  \|  \| \|  \| \|  \| \|  \| \|  \| \| \|  \|  \|  \|  \|  \|  \| fl 19 \| 71.3 \| 102.5 \| 103.6 \| \| 71.5 \| 6.3 \|  \|  \| \|  \| \|  \| \|  \| \|  \| \| \|  \|  \|  \|  \|  \|  \| fl 20 \| 71.3 \| 102.5 \| 83.7 \| \| 74.8 \| 7.4 \|  \|  \| \|  \| \|  \| \|  \| \|  \| \| \|  \|  \|  \|  \|  \|  \| fl 21 \| 71.3 \| 102.5 \| 96.5 \| \| 72.2 \| 5.3 \|  \|  \| \|  \| \|  \| \|  \| \|  \| \| \|  \|  \|  \|  \|  \|  \| fl 21 \| 71.3 \| 102.5 \| 133.4 \| \| 79.1 \| 4.8 \|  \|  \| \|  \| \|  \| \|  \| \|  \| \| \|  \|  \|  \|  \|  \|  \| flow 22 \| 71.3 \| 102.5 \| 120.2 \| \| 79.3 \| 4.5 \|  \|  \| \|  \| \|  \| \|  \| \|  \| \| \|  \|  \|  \|  \|  \|  \| fl 23 \| 71.3 \| 102.5 \| 132.5 \| \| 77.2 \| 3.2 \|  \|  \| \|  \| \|  \| \|  \| \|  \| \| \|  \|  \|  \|  \|  \|  \| fl 24 \| 71.3 \| 102.5 \| 103.9 \| \| 72.4 \| 4.6 \|  \|  \| \|  \| \|  \| \|  \| \|  \| \| \|  \|  \|  \|  \|  \|  \| fl 25 \| 71.3 \| 102.5 \| 126.2 \| \| 76.0 \| 5.0 \|  \|  \| \|  \| \|  \| \|  \| \|  \| \| \|  \|  \|  \|  \|  \|  \| fl 26 \| 71.3 \| 102.5 \| 118.5 \| \| 71.8 \| 4.8 \|  \|  \| \|  \| \|  \| \|  \| \|  \| \| \|  \|  \|  \|  \|  \|  \| fl 27 \| 71.3 \| 102.5 \| 112.9 \| \| 68.1 \| 5.3 \|  \|  \| \|  \| \|  \| \|  \| \|  \| \| \|  \|  \|  \|  \|  \|  \| fl 28 \| 71.3 \| 102.5 \| 134.9 \| \| 72.2 \| 4.4 \|  \|  \| \|  \| \|  \| \|  \| \|  \| \| \|  \|  \|  \|  \|  \|  \| fl 29 \| 71.3 \| 102.5 \| 130.8 \| \| 73.4 \| 3.4 \|  \|  \| \|  \| \|  \| \|  \| \|  \| \| \|  \|  \|  \|  \|  \|  \| flow 30 \| 71.3 \| 102.5 \| 142.3 \| \| 73.3 \| 6.5 \|  \|  \| \|  \| \|  \| \|  \| \|  \| \| \|  \|  \|  \|  \|  \|  \| fl 31 \| 71.3 \| 102.5 \| 141.6 \| \| 68.4 \| 5.5 \|  \|  \| \|  \| \|  \| \|  \| \|  \| \| \|  \|  \|  \|  \|  \|  \| fl 32 \| 71.3 \| 102.5 \| 118.3 \| \| 68.6 \| 4.6 \|  \|  \| \|  \| \|  \| \|  \| \|  \| \| \|  \|  \|  \|  \|  \|  \| fl 33 \| 71.3 \| 102.5 \| 144.1 \| \| 65.0 \| 6.8 \|  \|  \| \|  \| \|  \| \|  \| \|  \| \| \|  \|  \|  \|  \|  \|  \| fl 34 \| 71.3 \| 102.5 \| 115.2 \| \| 69.8 \| 2.4 \|  \|  \| \|  \| \|  \| \|  \| \|  \| \| \|  \|  \|  \|  \|  \|  \| fl 35 \| 71.3 \| 102.5 \| 128.9 \| \| 70.9 \| 7.6 \|  \|  \| \|  \| \|  \| \|  \| \|  \| \| \|  \|  \|  \|  \|  \|  \| flow 36 \| 71.3 \| 102.5 \| 115.7 \| \| 68.6 \| 5.0 \|  \|  \| \|  \| \|  \| \|  \| \|  \| \| \|  \|  \|  \|  \|  \|  \| flow 37 \| 71.3 \| 102.5 \| 129.1 \| \| 69.2 \| 6.0 \|  \|  \| \|  \| \|  \| \|  \| \|  \| \| \|  \|  \|  \|  \|  \|  \| fl 38 \| 71.3 \| 102.5 \| 131.1 \| \| 69.6 \| 4.8 \|  \|  \| \|  \| \|  \| \|  \| \|  \| \| \|  \|  \|  \|  \|  \|  \| fl 39 \| 71.3 \| 102.5 \| 126.7 \| \| 70.2 \| 5.4 \|  \|  \| \|  \| \|  \| \|  \| \|  \| \| \|  \|  \|  \|  \|  \|  \| fl 40 \| 71.3 \| 102.5 \| 116.8 \| \| 68.6 \| 4.1 \|  \|  \| \|  \| \|  \| \|  \| \|  \| \| \|  \|  \|  \|  \| \|  \|  \|  \|  \| **mean α_95_  (°):** \| \| \| 7.0 \|  \|  \| \|  \| \|  \| \|  \| \|  \| \| |
| --- | --- | --- | --- | --- | --- | --- | --- | --- | --- | --- | --- | --- | --- | --- | --- | --- | --- | --- | --- | --- | --- | --- | --- | --- | --- | --- | --- | --- | --- | --- | --- | --- | --- | --- | --- | --- | --- | --- | --- | --- | --- | --- | --- | --- | --- | --- | --- | --- | --- | --- | --- | --- | --- | --- | --- | --- | --- | --- | --- | --- | --- | --- | --- | --- | --- | --- | --- | --- | --- | --- | --- | --- | --- | --- | --- | --- | --- | --- | --- | --- | --- | --- | --- | --- | --- | --- | --- | --- | --- | --- | --- | --- | --- | --- | --- | --- | --- | --- | --- | --- | --- | --- | --- | --- | --- | --- | --- | --- | --- | --- | --- | --- | --- | --- | --- | --- | --- | --- | --- | --- | --- | --- | --- | --- | --- | --- | --- | --- | --- | --- | --- | --- | --- | --- | --- | --- | --- | --- | --- | --- | --- | --- | --- | --- | --- | --- | --- | --- | --- | --- | --- | --- | --- | --- | --- | --- | --- | --- | --- | --- | --- | --- | --- | --- | --- | --- | --- | --- | --- | --- | --- | --- | --- | --- | --- | --- | --- | --- | --- | --- | --- | --- | --- | --- | --- | --- | --- | --- | --- | --- | --- | --- | --- | --- | --- | --- | --- | --- | --- | --- | --- | --- | --- | --- | --- | --- | --- | --- | --- | --- | --- | --- | --- | --- | --- | --- | --- | --- | --- | --- | --- | --- | --- | --- | --- | --- | --- | --- | --- | --- | --- | --- | --- | --- | --- | --- | --- | --- | --- | --- | --- | --- | --- | --- | --- | --- | --- | --- | --- | --- | --- | --- | --- | --- | --- | --- | --- | --- | --- | --- | --- | --- | --- | --- | --- | --- | --- | --- | --- | --- | --- | --- | --- | --- | --- | --- | --- | --- | --- | --- | --- | --- | --- | --- | --- | --- | --- | --- | --- | --- | --- | --- | --- | --- | --- | --- | --- | --- | --- | --- | --- | --- | --- | --- | --- | --- | --- | --- | --- | --- | --- | --- | --- | --- | --- | --- | --- | --- | --- | --- | --- | --- | --- | --- | --- | --- | --- | --- | --- | --- | --- | --- | --- | --- | --- | --- | --- | --- | --- | --- | --- | --- | --- | --- | --- | --- | --- | --- | --- | --- | --- | --- | --- | --- | --- | --- | --- | --- | --- | --- | --- | --- | --- | --- | --- | --- | --- | --- | --- | --- | --- | --- | --- | --- | --- | --- | --- | --- | --- | --- | --- | --- | --- | --- | --- | --- | --- | --- | --- | --- | --- | --- | --- | --- | --- | --- | --- | --- | --- | --- | --- | --- | --- | --- | --- | --- | --- | --- | --- | --- | --- | --- | --- | --- | --- | --- | --- | --- | --- | --- | --- | --- | --- | --- | --- | --- | --- | --- | --- | --- | --- | --- | --- | --- | --- | --- | --- | --- | --- | --- | --- | --- | --- | --- | --- | --- | --- | --- | --- | --- | --- | --- | --- | --- | --- | --- | --- | --- | --- | --- | --- | --- | --- | --- | --- | --- | --- | --- | --- | --- | --- | --- | --- | --- | --- | --- | --- | --- | --- | --- | --- | --- | --- | --- | --- | --- | --- | --- | --- | --- | --- | --- | --- | --- | --- | --- | --- | --- | --- | --- | --- | --- | --- | --- | --- | --- | --- | --- | --- | --- | --- | --- | --- | --- | --- | --- | --- | --- | --- | --- | --- | --- | --- | --- | --- | --- | --- | --- | --- | --- | --- | --- | --- | --- | --- | --- | --- | --- | --- | --- | --- | --- | --- | --- | --- | --- | --- | --- | --- | --- | --- | --- | --- | --- | --- | --- | --- | --- | --- | --- | --- | --- | --- | --- | --- | --- | --- | --- | --- | --- | --- | --- | --- | --- | --- | --- | --- | --- | --- | --- | --- | --- | --- | --- | --- | --- | --- | --- | --- | --- | --- | --- | --- | --- | --- | --- | --- | --- | --- | --- | --- | --- | --- | --- | --- | --- | --- | --- | --- | --- | --- | --- | --- | --- | --- | --- | --- | --- | --- | --- | --- | --- | --- | --- | --- | --- | --- | --- | --- | --- | --- | --- | --- | --- | --- | --- | --- | --- | --- | --- | --- | --- | --- | --- | --- | --- | --- | --- | --- | --- | --- | --- | --- | --- | --- | --- | --- | --- | --- | --- | --- | --- | --- | --- | --- | --- | --- | --- | --- | --- | --- | --- | --- | --- | --- | --- | --- | --- | --- | --- | --- | --- | --- | --- | --- | --- | --- | --- | --- | --- | --- | --- | --- | --- | --- | --- | --- | --- | --- | --- | --- | --- | --- | --- | --- | --- | --- | --- | --- | --- | --- | --- | --- | --- | --- | --- | --- | --- | --- | --- | --- | --- | --- | --- | --- | --- | --- | --- | --- | --- | --- | --- | --- | --- | --- | --- | --- | --- | --- | --- | --- | --- | --- | --- | --- | --- | --- | --- | --- | --- | --- | --- | --- | --- | --- | --- | --- | --- | --- | --- | --- | --- | --- | --- | --- | --- | --- | --- | --- | --- | --- | --- | --- | --- | --- | --- | --- | --- | --- | --- | --- | --- | --- | --- | --- | --- | --- | --- | --- | --- | --- | --- | --- | --- | --- | --- | --- | --- | --- | --- | --- | --- | --- | --- | --- | --- | --- | --- | --- | --- | --- | --- | --- | --- | --- | --- | --- | --- | --- | --- | --- | --- | --- | --- | --- | --- | --- | --- | --- | --- | --- | --- | --- | --- | --- | --- | --- | --- | --- | --- | --- | --- | --- | --- | --- | --- | --- | --- | --- | --- | --- | --- | --- | --- | --- | --- | --- | --- | --- | --- | --- | --- | --- | --- | --- | --- | --- | --- | --- | --- | --- | --- | --- | --- | --- | --- | --- | --- | --- | --- | --- | --- | --- | --- | --- | --- | --- | --- | --- | --- | --- | --- | --- | --- | --- | --- | --- | --- | --- | --- | --- | --- | --- | --- | --- | --- | --- | --- | --- | --- | --- | --- | --- | --- | --- | --- | --- | --- | --- | --- | --- | --- | --- | --- | --- | --- | --- | --- | --- | --- | --- | --- | --- | --- | --- | --- | --- | --- | --- | --- | --- | --- | --- | --- | --- | --- | --- | --- | --- | --- | --- | --- | --- | --- | --- | --- | --- | --- | --- | --- | --- | --- | --- | --- | --- | --- | --- | --- | --- | --- | --- | --- | --- | --- | --- | --- | --- | --- | --- | --- | --- | --- | --- | --- | --- | --- | --- | --- | --- | --- | --- | --- | --- | --- | --- | --- | --- | --- | --- | --- | --- | --- | --- | --- | --- | --- | --- | --- | --- | --- | --- | --- | --- | --- | --- | --- | --- | --- | --- | --- | --- | --- | --- | --- | --- | --- | --- | --- | --- | --- | --- | --- | --- | --- | --- | --- | --- | --- | --- | --- | --- | --- | --- | --- | --- | --- | --- | --- | --- | --- | --- | --- | --- | --- | --- | --- | --- | --- | --- | --- | --- | --- | --- | --- | --- | --- | --- | --- | --- | --- | --- | --- | --- | --- | --- | --- | --- | --- | --- | --- | --- | --- | --- | --- | --- | --- | --- | --- | --- | --- | --- | --- | --- | --- | --- | --- | --- | --- | --- | --- | --- | --- | --- | --- | --- | --- | --- | --- | --- | --- | --- | --- | --- | --- | --- | --- | --- | --- | --- | --- | --- | --- | --- | --- | --- | --- | --- | --- | --- | --- | --- | --- | --- | --- | --- | --- | --- | --- | --- | --- | --- | --- | --- | --- | --- | --- | --- | --- | --- | --- | --- | --- | --- | --- | --- | --- | --- | --- | --- | --- | --- | --- | --- | --- | --- | --- | --- | --- | --- | --- | --- | --- | --- | --- | --- | --- | --- | --- | --- | --- | --- | --- | --- | --- | --- | --- | --- | --- | --- | --- | --- | --- | --- | --- | --- | --- | --- | --- | --- | --- | --- | --- | --- | --- | --- | --- | --- | --- | --- | --- | --- | --- | --- | --- | --- | --- | --- | --- | --- | --- | --- | --- | --- | --- | --- | --- | --- | --- | --- | --- | --- | --- | --- | --- | --- | --- | --- | --- | --- | --- | --- | --- | --- | --- | --- | --- | --- | --- | --- | --- | --- | --- | --- | --- | --- | --- | --- | --- | --- | --- | --- | --- | --- | --- | --- | --- | --- | --- | --- | --- | --- | --- | --- | --- | --- | --- | --- | --- | --- | --- | --- | --- | --- | --- | --- | --- | --- | --- | --- | --- | --- | --- | --- | --- | --- | --- | --- | --- | --- | --- | --- | --- | --- | --- | --- | --- | --- | --- | --- | --- | --- | --- | --- | --- | --- | --- | --- | --- | --- | --- | --- | --- | --- | --- | --- | --- | --- | --- | --- | --- | --- | --- | --- | --- | --- | --- | --- | --- | --- | --- | --- | --- | --- | --- | --- | --- | --- | --- | --- | --- | --- | --- | --- | --- | --- | --- | --- | --- | --- | --- | --- | --- | --- | --- | --- | --- | --- | --- | --- | --- | --- | --- | --- | --- | --- | --- | --- | --- | --- | --- | --- | --- | --- | --- | --- | --- | --- | --- | --- | --- | --- | --- | --- | --- | --- | --- | --- | --- | --- | --- | --- | --- | --- | --- | --- | --- | --- | --- | --- | --- | --- | --- | --- | --- | --- | --- | --- | --- | --- | --- | --- | --- | --- | --- | --- | --- | --- | --- | --- | --- | --- | --- | --- | --- | --- | --- | --- | --- | --- | --- | --- | --- | --- | --- | --- | --- | --- | --- | --- | --- | --- | --- | --- | --- | --- | --- | --- | --- | --- | --- | --- | --- | --- | --- | --- | --- | --- | --- | --- | --- | --- | --- | --- | --- | --- | --- | --- | --- | --- | --- | --- | --- | --- | --- | --- | --- | --- | --- | --- | --- | --- | --- | --- | --- | --- | --- | --- | --- | --- | --- | --- | --- | --- | --- | --- | --- | --- | --- | --- | --- | --- | --- | --- | --- | --- | --- | --- | --- | --- | --- | --- | --- | --- | --- | --- | --- | --- | --- | --- | --- | --- | --- | --- | --- | --- | --- | --- | --- | --- | --- | --- | --- | --- | --- | --- | --- | --- | --- | --- | --- | --- | --- | --- | --- | --- | --- | --- | --- | --- | --- | --- | --- | --- | --- | --- | --- | --- | --- | --- | --- | --- | --- | --- | --- | --- | --- | --- | --- | --- | --- | --- | --- | --- | --- | --- | --- | --- | --- | --- | --- | --- | --- | --- | --- | --- | --- | --- | --- | --- | --- | --- | --- | --- | --- | --- | --- | --- | --- | --- | --- | --- | --- | --- | --- | --- | --- | --- | --- | --- | --- | --- | --- | --- | --- | --- | --- | --- | --- | --- | --- | --- | --- | --- | --- | --- | --- | --- | --- | --- | --- | --- | --- | --- | --- | --- | --- | --- | --- | --- | --- | --- | --- | --- | --- | --- | --- | --- | --- | --- | --- | --- | --- | --- | --- | --- | --- | --- | --- | --- | --- | --- | --- | --- | --- | --- | --- | --- | --- | --- | --- | --- | --- | --- | --- | --- | --- | --- | --- | --- | --- | --- | --- | --- | --- | --- | --- | --- | --- | --- | --- | --- | --- | --- | --- | --- | --- | --- | --- | --- | --- | --- | --- | --- | --- | --- | --- | --- | --- | --- | --- | --- | --- | --- | --- | --- | --- | --- | --- | --- | --- | --- | --- | --- | --- | --- | --- | --- | --- | --- | --- | --- | --- | --- | --- | --- | --- | --- | --- | --- | --- | --- | --- | --- | --- | --- | --- | --- | --- | --- | --- | --- | --- | --- | --- | --- | --- | --- | --- | --- | --- | --- | --- | --- | --- | --- | --- | --- | --- | --- | --- | --- | --- | --- | --- | --- | --- | --- | --- | --- | --- | --- | --- | --- | --- | --- | --- | --- | --- | --- | --- | --- | --- | --- | --- | --- | --- | --- | --- | --- | --- | --- | --- | --- | --- | --- | --- | --- | --- | --- | --- | --- | --- | --- | --- | --- | --- | --- | --- | --- | --- |

| \| **Supplementary Table 1 (continuation):** Directional and statistical data (site latitude (λ_S_) and longitude (ϕ_S_); declination (D_ChRM_) and inclination (I_ChRM_) of characteristic remanent magnetization; α_95_: 95% confidence cone) from the selected paleomagnetic database (according to the selection criteria discussed at section 2.1). Numbering of the datasets (*) are in accordance to Table 1. References are listed in "References (Table 1)" worksheet. \| \| \| \| \| \| \| \| \| \| \| \| \| \| \| \| \| \| \| --- \| --- \| --- \| --- \| --- \| --- \| --- \| --- \| --- \| --- \| --- \| --- \| --- \| --- \| --- \| --- \| --- \| --- \| \| **(7) Gurevitch et al. (2004) *** \| \| \| \| \| \| **(8) Heunemann et al. (2004) *** \| \| \| \| \| \| **(9) Kravchinsky et al. (2002) *** \| \| \| \| \| \| \| **Site** \| **λ_s_ (°N)** \| **ϕ_s_ (°E)** \| **D_ChRM_ (°)** \| **I_ChRM_ (°)** \| **α_95_  (°)** \| **Site** \| **λ_s_ (°N)** \| **ϕ_s_ (°E)** \| **D_ChRM_ (°)** \| **I_ChRM_ (°)** \| **α_95_  (°)** \| **Site** \| **λ_s_ (°N)** \| **ϕ_s_ (°E)** \| **D_ChRM_ (°)** \| **I_ChRM_ (°)** \| **α_95_  (°)** \| \| sm \| 70.0 \| 88.5 \| 81.3 \| 69.5 \| - \| ND6 \| 70.37 \| 90.01 \| 14.0 \| 69.0 \| 2.2 \| Stk-1 \| 66.11 \| 111.80 \| 275.9 \| -59.9 \| 5.0 \| \| km \| 70.0 \| 88.5 \| 63.2 \| 73.1 \| 4.6 \| ND7 \| 70.37 \| 90.01 \| 04.0 \| 72.0 \| 2.2 \| Stk-2 \| 66.11 \| 111.80 \| 262.6 \| -67.0 \| 6.8 \| \| hr \| 70.0 \| 88.5 \| 94.3 \| 68.7 \| 10.4 \| ND8 \| 70.37 \| 90.01 \| 35.0 \| 69.0 \| 2.8 \| Stk-3 \| 66.11 \| 111.80 \| 279.1 \| -71.0 \| 7.9 \| \| mk \| 70.0 \| 88.5 \| 95.4 \| 74.2 \| 4.5 \| ND9 \| 70.37 \| 90.01 \| 28.0 \| 67.0 \| 2.8 \| Stk-4 \| 66.11 \| 111.80 \| 278.7 \| -61.8 \| 10.9 \| \| mr \| 70.0 \| 88.5 \| 90.5 \| 80.0 \| 3.6 \| ND10 \| 70.37 \| 90.01 \| 18.0 \| 69.0 \| 3.6 \| Stk-5 \| 66.11 \| 111.80 \| 284.8 \| -61.8 \| 26.7 \| \| nd \| 70.0 \| 88.5 \| 28.1 \| 70.6 \| 4.6 \| ND11 \| 70.37 \| 90.01 \| 07.0 \| 66.0 \| 3.2 \| Stk-6 \| 66.11 \| 111.80 \| 277.6 \| -64.5 \| 5.7 \| \| tk \| 70.0 \| 88.5 \| 36.9 \| 74.0 \| 7.8 \| ND12 \| 70.37 \| 90.01 \| 14.0 \| 66.0 \| 2.6 \| Stk-7 \| 66.11 \| 111.80 \| 266.8 \| -67.1 \| 22.5 \| \| hk \| 70.0 \| 88.5 \| 90.3 \| 57.6 \| - \| ND13 \| 70.37 \| 90.01 \| 10.0 \| 69.0 \| 4.0 \| Stk-8 \| 66.11 \| 111.80 \| 266.5 \| -59.4 \| 14.7 \| \| gd \| 70.0 \| 88.5 \| 107.3 \| 66.0 \| 11.0 \| ND14 \| 70.37 \| 90.01 \| 20.0 \| 65.0 \| 5.1 \| Stk-9 \| 66.11 \| 111.80 \| 254.9 \| -62.9 \| 17.2 \| \| sv \| 70.0 \| 88.5 \| 150.9 \| 52.6 \| 2.7 \| ND25 \| 70.37 \| 90.01 \| 31.0 \| 64.0 \| 9.2 \| Stk-10 \| 66.11 \| 111.80 \| 285.1 \| -62.7 \| 12.9 \| \| iv (R) \| 70.0 \| 88.5 \| 55.5 \| 70.9 \| 9.1 \| ND26 \| 70.37 \| 90.01 \| 38.0 \| 62.0 \| 2.7 \|  \|  \|  \| **mean α_95_  (°):** \| \| 13.0 \| \| iv (N) \| 70.0 \| 88.5 \| 150.5 \| 66.7 \| 15.8 \| TK3 \| 70.37 \| 90.01 \| 26.0 \| 69.0 \| 7.2 \|  \|  \|  \|  \|  \|  \| \|  \|  \|  \| **mean α_95_  (°):** \| \| 6.2 \| TK4 \| 70.37 \| 90.01 \| 23.0 \| 75.0 \| 3.7 \|  \|  \|  \|  \| \|  \| \|  \|  \|  \|  \|  \|  \| TK5 \| 70.37 \| 90.01 \| 35.0 \| 70.0 \| 5.9 \|  \|  \|  \|  \|  \|  \| \|  \|  \|  \|  \|  \|  \|  \|  \|  \| **mean α_95_  (°):** \| \| 4.1 \|  \|  \|  \|  \|  \|  \| |
| --- | --- | --- | --- | --- | --- | --- | --- | --- | --- | --- | --- | --- | --- | --- | --- | --- | --- | --- | --- | --- | --- | --- | --- | --- | --- | --- | --- | --- | --- | --- | --- | --- | --- | --- | --- | --- | --- | --- | --- | --- | --- | --- | --- | --- | --- | --- | --- | --- | --- | --- | --- | --- | --- | --- | --- | --- | --- | --- | --- | --- | --- | --- | --- | --- | --- | --- | --- | --- | --- | --- | --- | --- | --- | --- | --- | --- | --- | --- | --- | --- | --- | --- | --- | --- | --- | --- | --- | --- | --- | --- | --- | --- | --- | --- | --- | --- | --- | --- | --- | --- | --- | --- | --- | --- | --- | --- | --- | --- | --- | --- | --- | --- | --- | --- | --- | --- | --- | --- | --- | --- | --- | --- | --- | --- | --- | --- | --- | --- | --- | --- | --- | --- | --- | --- | --- | --- | --- | --- | --- | --- | --- | --- | --- | --- | --- | --- | --- | --- | --- | --- | --- | --- | --- | --- | --- | --- | --- | --- | --- | --- | --- | --- | --- | --- | --- | --- | --- | --- | --- | --- | --- | --- | --- | --- | --- | --- | --- | --- | --- | --- | --- | --- | --- | --- | --- | --- | --- | --- | --- | --- | --- | --- | --- | --- | --- | --- | --- | --- | --- | --- | --- | --- | --- | --- | --- | --- | --- | --- | --- | --- | --- | --- | --- | --- | --- | --- | --- | --- | --- | --- | --- | --- | --- | --- | --- | --- | --- | --- | --- | --- | --- | --- | --- | --- | --- | --- | --- | --- | --- | --- | --- | --- | --- | --- | --- | --- | --- | --- | --- | --- | --- | --- | --- | --- | --- | --- | --- | --- | --- | --- | --- | --- | --- | --- | --- | --- | --- | --- | --- | --- | --- | --- | --- | --- | --- | --- | --- | --- | --- | --- | --- | --- | --- | --- | --- | --- | --- | --- | --- | --- | --- | --- | --- | --- | --- | --- | --- | --- | --- | --- | --- | --- | --- | --- | --- | --- | --- | --- | --- | --- | --- | --- | --- | --- | --- | --- | --- | --- | --- | --- | --- | --- | --- | --- |

| \| **Supplementary Table 1 (continuation):** Directional and statistical data (site latitude (λ_S_) and longitude (ϕ_S_); declination (D_ChRM_) and inclination (I_ChRM_) of characteristic remanent magnetization; α_95_: 95% confidence cone) from the selected paleomagnetic database (according to the selection criteria discussed at section 2.1). Numbering of the datasets (*) are in accordance to Table 1. References are listed in "References (Table 1)" worksheet. \| \| \| \| \| \| \| \| \| \| \| \| \| \| \| \| \| \| \| \| --- \| --- \| --- \| --- \| --- \| --- \| --- \| --- \| --- \| --- \| --- \| --- \| --- \| --- \| --- \| --- \| --- \| --- \| --- \| \| **(10) Heunemann et al. (2004) *** \| \| \| \| \| \| **(11) Van der Voo et al. (1993) *** \| \| \| \| \| \| **(12) Yokoyama et al. (2014) *** \| \| \| \| \| \| \| \| **Site** \| **λ_s_ (°N)** \| **ϕ_s_ (°E)** \| **D_ChRM_ (°)** \| **I_ChRM_ (°)** \| **α_95_  (°)** \| **Site** \| **λ_s_ (°N)** \| **ϕ_s_ (°E)** \| **D_ChRM_ (°)** \| **I_ChRM_ (°)** \| **α_95_  (°)** \| **Site** \| **λ_s_ (°N)** \| **ϕ_s_ (°E)** \| **D_ChRM_ (°)** \| \| **I_ChRM_ (°)** \| **α_95_  (°)** \| \| GD1 \| 69.47 \| 88.72 \| 145.0 \| 55.0 \| 5.9 \| 17 \| 25.0 \| 102.7 \| 31.4 \| -18.0 \| 4.3 \| 8 \| -16.78 \| -53.0 \| 355.7 \| \| -40.5 \| 3.9 \| \| SV11 \| 69.47 \| 88.72 \| 153.0 \| 58.0 \| 5.1 \| 18 \| 25.0 \| 102.7 \| 42.5 \| -8.6 \| 7.0 \| 14 \| -16.78 \| -53.0 \| 5.3 \| \| -27.0 \| 15.4 \| \| SV10 \| 69.47 \| 88.72 \| 151.0 \| 53.0 \| 3.0 \| 32 \| 25.0 \| 102.7 \| 34.0 \| -13.7 \| 6.5 \| 21 \| -16.78 \| -53.0 \| 358.1 \| \| -41.8 \| 3.1 \| \| SV9 \| 69.47 \| 88.72 \| 161.0 \| 50.0 \| 7.6 \| 90 \| 25.0 \| 102.7 \| 27.3 \| -15.9 \| 4.3 \| 22 \| -16.78 \| -53.0 \| 1.9 \| \| -44.4 \| 6.2 \| \| SV8 \| 69.47 \| 88.72 \| 155.0 \| 56.0 \| 5.9 \| 91 \| 25.0 \| 102.7 \| 28.7 \| -1.5 \| 5.0 \| 29 \| -16.78 \| -53.0 \| 9.0 \| \| -38.3 \| 2.3 \| \| SV7 \| 69.47 \| 88.72 \| 148.0 \| 56.0 \| 4.3 \| 92 \| 25.0 \| 102.7 \| 27.2 \| -8.2 \| 4.4 \| 30 \| -16.78 \| -53.0 \| 1.3 \| \| -41.1 \| 4.0 \| \| SV6 \| 69.47 \| 88.72 \| 152.0 \| 54.0 \| 5.6 \| 167 \| 25.0 \| 102.7 \| 17.2 \| -15.5 \| 4.7 \| 39 \| -16.78 \| -53.0 \| 5.3 \| \| -34.4 \| 2.9 \| \| SV5 \| 69.47 \| 88.72 \| 149.0 \| 54.0 \| 3.7 \| 168 \| 25.0 \| 102.7 \| 19.3 \| -15.2 \| 3.5 \| 40 \| -16.78 \| -53.0 \| 355.0 \| \| -38.4 \| 5.9 \| \| SV4 \| 69.47 \| 88.72 \| 153.0 \| 55.0 \| 5.4 \| 169 \| 25.0 \| 102.7 \| 8.6 \| -8.1 \| 6.6 \| 42 \| -16.78 \| -53.0 \| 6.5 \| \| -38.5 \| 10.0 \| \| SV3 \| 69.47 \| 88.72 \| 148.0 \| 48.0 \| 5.5 \| 171 \| 25.0 \| 102.7 \| 20.7 \| -10.8 \| 5.4 \| 43 \| -16.78 \| -53.0 \| 336.2 \| \| -37.6 \| 15.5 \| \| SV2 \| 69.47 \| 88.72 \| 148.0 \| 58.0 \| 6.8 \|  \|  \|  \| **mean α_95_  (°):** \| \| 5.2 \| 44 \| -16.78 \| -53.0 \| 347.5 \| -42.4 \| \| 3.8 \| \| SV1 \| 69.47 \| 88.72 \| 152.0 \| 55.0 \| 4.9 \|  \|  \|  \|  \|  \|  \| 46 \| -16.78 \| -53.0 \| 338.9 \| \| -45.6 \| 6.2 \| \| TA8 \| 69.47 \| 88.72 \| 146.0 \| 58.0 \| 5.4 \|  \|  \|  \|  \|  \|  \| 48 \| -16.78 \| -53.0 \| 357.7 \| \| -37.0 \| 5.7 \| \| TA7 \| 69.47 \| 88.72 \| 152.0 \| 49.0 \| 6.6 \|  \|  \|  \|  \|  \|  \| 61 \| -16.78 \| -53.0 \| 346.2 \| \| -44.3 \| 6.1 \| \| TA6 \| 69.47 \| 88.72 \| 166.0 \| 56.0 \| 13.8 \|  \|  \|  \|  \|  \|  \| 62 \| -16.78 \| -53.0 \| 352.1 \| \| -39.0 \| 4.4 \| \|  \|  \|  \| **mean α_95_  (°):** \| \| 6.0 \|  \|  \|  \|  \|  \|  \| 67 \| -16.78 \| -53.0 \| 359.2 \| \| -30.7 \| 14.8 \| \|  \|  \|  \|  \|  \|  \|  \|  \|  \|  \|  \|  \| 68 \| -16.78 \| -53.0 \| 353.1 \| \| -29.2 \| 6.7 \| \|  \|  \|  \|  \|  \|  \|  \|  \|  \|  \|  \|  \| 71 \| -16.78 \| -53.0 \| 359.1 \| \| -35.5 \| 9.9 \| \|  \|  \|  \|  \|  \|  \|  \|  \|  \|  \|  \|  \| 73 \| -16.78 \| -53.0 \| 353.7 \| \| -37.2 \| 2.1 \| \|  \|  \|  \|  \|  \|  \|  \|  \|  \|  \|  \|  \| 78 \| -16.78 \| -53.0 \| 8.3 \| \| -42.6 \| 5.0 \| \|  \|  \|  \|  \|  \|  \|  \|  \|  \|  \|  \|  \| 79 \| -16.78 \| -53.0 \| 350.8 \| \| -40.8 \| 10.0 \| \|  \|  \|  \|  \|  \|  \|  \|  \|  \|  \|  \|  \| G-1 \| -16.78 \| -53.0 \| 7.1 \| \| -40.6 \| 8.8 \| \|  \|  \|  \|  \|  \|  \|  \|  \|  \|  \|  \|  \| G-2 \| -16.78 \| -53.0 \| 18.4 \| \| -43.0 \| 13.5 \| \|  \|  \|  \|  \|  \|  \|  \|  \|  \|  \|  \|  \| G-3 \| -16.78 \| -53.0 \| 8.9 \| \| -40.0 \| 7.4 \| \|  \|  \|  \|  \|  \|  \|  \|  \|  \|  \|  \|  \| G-4 \| -16.78 \| -53.0 \| 3.8 \| \| -37.5 \| 12.7 \| \|  \|  \|  \|  \|  \|  \|  \|  \|  \|  \|  \|  \| G-5 \| -16.78 \| -53.0 \| 9.3 \| \| -39.7 \| 8.8 \| \|  \|  \|  \|  \|  \|  \|  \|  \|  \|  \|  \|  \| G-6 \| -16.78 \| -53.0 \| 323.1 \| \| -29.1 \| 7.8 \| \|  \|  \|  \|  \|  \|  \|  \|  \|  \|  \| \|  \|  \|  \|  \| **mean α_95_  (°):** \| \| \| 7.5 \| |
| --- | --- | --- | --- | --- | --- | --- | --- | --- | --- | --- | --- | --- | --- | --- | --- | --- | --- | --- | --- | --- | --- | --- | --- | --- | --- | --- | --- | --- | --- | --- | --- | --- | --- | --- | --- | --- | --- | --- | --- | --- | --- | --- | --- | --- | --- | --- | --- | --- | --- | --- | --- | --- | --- | --- | --- | --- | --- | --- | --- | --- | --- | --- | --- | --- | --- | --- | --- | --- | --- | --- | --- | --- | --- | --- | --- | --- | --- | --- | --- | --- | --- | --- | --- | --- | --- | --- | --- | --- | --- | --- | --- | --- | --- | --- | --- | --- | --- | --- | --- | --- | --- | --- | --- | --- | --- | --- | --- | --- | --- | --- | --- | --- | --- | --- | --- | --- | --- | --- | --- | --- | --- | --- | --- | --- | --- | --- | --- | --- | --- | --- | --- | --- | --- | --- | --- | --- | --- | --- | --- | --- | --- | --- | --- | --- | --- | --- | --- | --- | --- | --- | --- | --- | --- | --- | --- | --- | --- | --- | --- | --- | --- | --- | --- | --- | --- | --- | --- | --- | --- | --- | --- | --- | --- | --- | --- | --- | --- | --- | --- | --- | --- | --- | --- | --- | --- | --- | --- | --- | --- | --- | --- | --- | --- | --- | --- | --- | --- | --- | --- | --- | --- | --- | --- | --- | --- | --- | --- | --- | --- | --- | --- | --- | --- | --- | --- | --- | --- | --- | --- | --- | --- | --- | --- | --- | --- | --- | --- | --- | --- | --- | --- | --- | --- | --- | --- | --- | --- | --- | --- | --- | --- | --- | --- | --- | --- | --- | --- | --- | --- | --- | --- | --- | --- | --- | --- | --- | --- | --- | --- | --- | --- | --- | --- | --- | --- | --- | --- | --- | --- | --- | --- | --- | --- | --- | --- | --- | --- | --- | --- | --- | --- | --- | --- | --- | --- | --- | --- | --- | --- | --- | --- | --- | --- | --- | --- | --- | --- | --- | --- | --- | --- | --- | --- | --- | --- | --- | --- | --- | --- | --- | --- | --- | --- | --- | --- | --- | --- | --- | --- | --- | --- | --- | --- | --- | --- | --- | --- | --- | --- | --- | --- | --- | --- | --- | --- | --- | --- | --- | --- | --- | --- | --- | --- | --- | --- | --- | --- | --- | --- | --- | --- | --- | --- | --- | --- | --- | --- | --- | --- | --- | --- | --- | --- | --- | --- | --- | --- | --- | --- | --- | --- | --- | --- | --- | --- | --- | --- | --- | --- | --- | --- | --- | --- | --- | --- | --- | --- | --- | --- | --- | --- | --- | --- | --- | --- | --- | --- | --- | --- | --- | --- | --- | --- | --- | --- | --- | --- | --- | --- | --- | --- | --- | --- | --- | --- | --- | --- | --- | --- | --- | --- | --- | --- | --- | --- | --- | --- | --- | --- | --- | --- | --- | --- | --- | --- | --- | --- | --- | --- | --- | --- | --- | --- | --- | --- | --- | --- | --- | --- | --- | --- | --- | --- | --- | --- | --- | --- | --- | --- | --- | --- | --- | --- | --- | --- | --- | --- | --- | --- | --- | --- | --- | --- | --- | --- | --- | --- | --- | --- | --- | --- | --- | --- | --- | --- | --- | --- | --- | --- | --- | --- | --- | --- | --- | --- | --- | --- | --- | --- | --- | --- | --- | --- | --- | --- | --- | --- | --- | --- | --- | --- | --- | --- | --- | --- | --- | --- | --- | --- | --- | --- | --- | --- | --- | --- | --- | --- | --- | --- | --- | --- | --- | --- | --- | --- | --- | --- | --- | --- | --- | --- | --- | --- | --- | --- | --- | --- | --- | --- | --- | --- | --- | --- | --- | --- | --- | --- | --- | --- | --- | --- | --- | --- | --- | --- | --- | --- | --- | --- | --- | --- | --- | --- | --- | --- | --- | --- | --- | --- | --- | --- | --- | --- | --- | --- | --- | --- | --- | --- |

| **Supplementary Table 1 (continuation):** Directional and statistical data (site latitude (λ_S_) and longitude (ϕ_S_); declination (D_ChRM_) and inclination (I_ChRM_) of characteristic remanent magnetization; α_95_: 95% confidence cone) from the selected paleomagnetic database (according to the selection criteria discussed at section 2.1). Numbering of the datasets (*) are in accordance to Table 1. References are listed in "References (Table 1)" worksheet. | | | | | | | | | | | | | | | | | | | |
| --- | --- | --- | --- | --- | --- | --- | --- | --- | --- | --- | --- | --- | --- | --- | --- | --- | --- | --- | --- |
| **(13) Tomezzoli et al. (2009) *** | | | | | | | **(14) Miguez et al. (2016) *** | | | | | | **(15) Domeier et al. (2011) *** | | | | | | |
| **Site** | **λ_s_ (°N)** | **ϕ_s_ (°E)** | **D_ChRM_ (°)** | **I_ChRM_ (°)** | | **α_95_  (°)** | **Site** | **λ_s_ (°N)** | **ϕ_s_ (°E)** | **D_ChRM_ (°)** | **I_ChRM_ (°)** | **α_95_  (°)** | **Site** | **λ_s_ (°N)** | **ϕ_s_ (°E)** | **D_ChRM_ (°)** | | **I_ChRM_ (°)** | **α_95_  (°)** |
| Sc1 | -38.0 | -65.5 | 146.0 | 43.0 | | 6.5 | DZ13 | -40.4 | -68.4 | 308.6 | -71.7 | 6.2 | RS16 | -37.9 | -65.45 | 165.2 | | 56.2 | 10.8 |
| Sc2 | -38.0 | -65.5 | 148.5 | 55.0 | | 6.0 | DZ15 | -40.4 | -68.4 | 352.4 | -65.6 | 3.4 | SC19 | -37.9 | -65.45 | 170.3 | | 47.0 | 3.0 |
| Sc3 | -38.0 | -65.5 | 170.0 | 46.0 | | 12.0 | DZ12 | -40.4 | -68.4 | 316.4 | -64.8 | 18.2 | RS15 | -37.9 | -65.45 | 174.5 | | 59.4 | 8.3 |
| Sc4 | -38.0 | -65.5 | 154.0 | 32.0 | | 13.0 | DZ2 | -40.4 | -68.4 | -352.3 | -55.2 | 9.4 | RS13 | -37.9 | -65.45 | 161.2 | | 58.2 | 4.4 |
| Sc5 | -38.0 | -65.5 | 161.0 | 56.0 | | 4.0 | DZ11 | -40.4 | -68.4 | 346.1 | -53.6 | 9.9 | SC18 | -37.9 | -65.45 | 157.4 | | 62.3 | 6.0 |
| Sc6 | -38.0 | -65.5 | 147.0 | 45.0 | | 8.0 | DZ3 | -40.4 | -68.4 | -355.2 | -51.1 | 13.6 | SC25 | -37.9 | -65.45 | 164.1 | | 62.0 | 6.6 |
| Sc7 | -38.0 | -65.5 | 151.0 | 30.0 | | 8.0 | DZ6 | -40.4 | -68.4 | -334.5 | -51.0 | 7.3 | RS12 | -37.9 | -65.45 | 164.6 | | 63.0 | 12.4 |
| Sc8 | -38.0 | -65.5 | 111.0 | 54.0 | | 4.0 | DZ16 | -40.4 | -68.4 | 334.6 | -43.6 | 14.2 | SC16 | -37.9 | -65.45 | 178.0 | | 58.1 | 2.3 |
| Sc9 | -38.0 | -65.5 | 136.5 | 80.0 | | 10.0 | DZ5 | -40.4 | -68.4 | -359.6 | -39.0 | 7.6 | SC24 | -37.9 | -65.45 | 175.2 | | 62.6 | 2.8 |
| Sc10 | -38.0 | -65.5 | 127.0 | 81.0 | | 8.0 | DZ10 | -40.4 | -68.4 | 354.1 | -51.9 | 4.3 | SC13 | -37.9 | -65.45 | 179.6 | | 55.3 | 2.6 |
|  |  |  | **mean α_95_  (°):** | | | 8.0 | DZ1 | -40.4 | -68.4 | 335.3 | -60.0 | 5.8 | RS11 | -37.9 | -65.45 | 169.6 | 46.9 | | 5.4 |
|  |  |  |  | |  |  | DZ9 | -40.4 | -68.4 | 348.1 | -63.3 | 9.7 | SC15 | -37.9 | -65.45 | 178.0 | | 58.6 | 4.7 |
|  |  |  |  | |  |  | DZ14 | -40.4 | -68.4 | -341.7 | -76.2 | 10.8 | SC14 | -37.9 | -65.45 | 169.5 | | 60.2 | 3.5 |
|  |  |  |  | |  |  |  |  |  | **mean α_95_  (°):** | | 9.3 | 61 | RS10 | -37.9 | -65.45 | | 166.4 | 61.8 |
|  |  |  |  | |  |  |  |  |  |  |  |  | SC22 | -37.9 | -65.45 | 164.1 | | -62.2 | 12.5 |
|  |  |  |  | | |  |  |  |  |  |  |  | 67 | SC23 | -37.9 | -65.45 | | 146.7 | 64.8 |
|  |  |  |  | |  |  |  |  |  |  |  |  | RS09 | -37.9 | -65.45 | 196.5 | | 53.4 | 4.5 |
|  |  |  |  | |  |  |  |  |  |  |  |  | SC11 | -37.9 | -65.45 | 182.1 | | 61.1 | 9.7 |
|  |  |  |  | |  |  |  |  |  |  |  |  | RS08 | -37.9 | -65.45 | 173.8 | | 71.0 | 12.2 |
|  |  |  |  | |  |  |  |  |  |  |  |  | SC21 | -37.9 | -65.45 | 163.2 | | 68.7 | 6.1 |
|  |  |  |  | |  |  |  |  |  |  |  |  | RS07 | -37.9 | -65.45 | 155.3 | | 65.3 | 8.9 |
|  |  |  |  | |  |  |  |  |  |  |  |  | RS06 | -37.9 | -65.45 | 160.8 | | 66.2 | 11.6 |
|  |  |  |  | |  |  |  |  |  |  |  |  | RS05 | -37.9 | -65.45 | 155.9 | | 63.7 | 15.4 |
|  |  |  |  | |  |  |  |  |  |  |  |  | SC08 | -37.9 | -65.45 | 179.5 | | 62.4 | 6.6 |
|  |  |  |  | |  |  |  |  |  |  |  |  | SC10 | -37.9 | -65.45 | 182.9 | | 60.9 | 11.9 |
|  |  |  |  | |  |  |  |  |  |  |  |  | RS03 | -37.9 | -65.45 | 156.2 | | 59.3 | 10.7 |
|  |  |  |  | |  |  |  |  |  |  |  |  | SC09 | -37.9 | -65.45 | 179.3 | | 67.8 | 4.8 |
|  |  |  |  | |  |  |  |  |  |  |  |  | RS04 | -37.9 | -65.45 | 167.0 | | 79.6 | 9.7 |
|  |  |  |  | |  |  |  |  |  |  |  |  | SC07 | -37.9 | -65.45 | 184.5 | | 55.2 | 5.4 |
|  |  |  |  | |  |  |  |  |  |  |  |  | SC06 | -37.9 | -65.45 | 166.6 | | 54.9 | 5.1 |
|  |  |  |  | |  |  |  |  |  |  |  |  | RS02 | -37.9 | -65.45 | 154.6 | | 68.5 | 12.5 |
|  |  |  |  | |  |  |  |  |  |  |  |  | RS17 | -37.9 | -65.45 | 344.7 | | -67.2 | 6.8 |
|  |  |  |  | |  |  |  |  |  |  |  |  | SC05 | -37.9 | -65.45 | 337.1 | | -62 | 2.7 |
|  |  |  |  | |  |  |  |  |  |  |  |  | SC20 | -37.9 | -65.45 | 353.8 | | -59.6 | 4.9 |
|  |  |  |  | |  |  |  |  |  |  |  |  | SC04 | -37.9 | -65.45 | 344 | | -50 | 3.0 |
|  |  |  |  | |  |  |  |  |  |  |  |  |  |  |  | **mean α_95_  (°):** | | | 6.9 |

| **Supplementary Table 1 (continuation):** Directional and statistical data (site latitude (λ_S_) and longitude (ϕ_S_); declination (D_ChRM_) and inclination (I_ChRM_) of characteristic remanent magnetization; α_95_: 95% confidence cone) from the selected paleomagnetic database (according to the selection criteria discussed at section 2.1). Numbering of the datasets (*) are in accordance to Table 1. References are listed in "References (Table 1)" worksheet. |
| --- |
| \| **(16) Belica et al. (2017) *** \| \| \| \| \| \| \| --- \| --- \| --- \| --- \| --- \| --- \| \| **Site** \| **λ_s_ (°N)** \| **ϕ_s_ (°E)** \| **D_ChRM_ (°)** \| **I_ChRM_ (°)** \| **α_95_  (°)** \| \| 1 \| -34.672 \| 150.858 \| 226.0 \| 82.6 \| 4.6 \| \| 2 \| -34.621 \| 150.845 \| 199.1 \| 78.9 \| 9.8 \| \| 3 \| -34.631 \| 150.850 \| 122.4 \| 72.9 \| 6.5 \| \| 4 \| -34.651 \| 150.853 \| 212.2 \| 86.6 \| 6.1 \| \| 5 \| -34.653 \| 150.856 \| 211.7 \| 75.7 \| 3.8 \| \| 6 \| -34.616 \| 150.828 \| 208.5 \| 77.8 \| 4.2 \| \| 7 \| -34.477 \| 150.914 \| 130.8 \| 77.8 \| 13.7 \| \| 8 \| -34.477 \| 150.914 \| 145.3 \| 66.9 \| 12.4 \| \| 9 \| -34.477 \| 150.914 \| 273.3 \| 85.6 \| 9.5 \| \| 10 \| -34.477 \| 150.914 \| 315.7 \| -79.2 \| 6.3 \| \| 11 \| -34.491 \| 150.914 \| 42.5 \| -70.2 \| 19.4 \| \| 12 \| -34.691 \| 150.823 \| 333.4 \| -68.3 \| 11.9 \| \| 13 \| -34.698 \| 150.824 \| 182.7 \| 70.3 \| 3.2 \| \| 14 \| -34.610 \| 150.756 \| 8.8 \| -71.9 \| 14.3 \| \| 15 \| -34.726 \| 150.654 \| 142.0 \| 57.9 \| 10.7 \| \| 16 \| -34.732 \| 150.687 \| 205.2 \| 70.2 \| 10.1 \| \| 17 \| -34.437 \| 150.837 \| 315.5 \| -83.3 \| 17.5 \| \|  \|  \|  \| **mean α_95_  (°):** \| \| 9.3 \| |
