# Supplementary material for: Paleomagnetic Evidence for Inverse Correspondence between the Relative Contribution of the Axial Dipole Field and CMB Heat Flux for the Past 270 Myr
Source: Sci Rep. 2019 Jan 22;9:282. doi: 10.1038/s41598-018-36494-x (PMC6342988; doi:10.1038/s41598-018-36494-x)
Supplement: Supplementary file 2 — Supplementary Table S2 [file 41598_2018_36494_MOESM2_ESM.docx]

Supplementary Information

**Paleomagnetic evidence for inverse correspondence between the relative contribution of the axial dipole field and CMB heat flux for the past 270 Myr**

Daniel Ribeiro Franco^1*^, Wellington Paulo de Oliveira^1^, Felipe Barbosa Venâncio de Freitas^1^, Diego Takahashi^1^, Cosme Ferreira da Ponte Neto^1^, Ian Muzy Camarão Peixoto^2^.

^1^Coordenação de Geofísica, Observatório Nacional, R. Gal. José Cristino, 77, 20921-400 Rio de Janeiro, RJ, Brazil,

^2^Instituto de Geociências, Universidade Federal Fluminense, Av. Milton Tavares de Souza, S/N, 24210-346 Niterói, RJ, Brazil

*To whom correspondence should be addressed. E-mail: drfranco@on.br

| **Supplementary Table 2:** Selected paleomagnetic database and its corresponding references listed in Tables 1 and S1. | |
| --- | --- |
| **Nr.** | **Study** |
| **16** | Belica, M. E., Tohver, E., Pisarevsky, S. A., Jourdan, F., Denyszyn, S., & George, A. D. Middle Permian paleomagnetism of the Sydney Basin, Eastern Gondwana: Testing Pangea models and the timing of the end of the Kiaman Reverse Superchron. *Tectonophysics*, **699**, 178-198 (2017). |
| **15** | Domeier, M., Van der Voo, R., Tohver, E., Tomezzoli, R. N., Vizan, H., Torsvik, T. H., & Kirshner, J. New Late Permian paleomagnetic data from Argentina: refinement of the apparent polar wander path of Gondwana. *Geochemistry, Geophysics, Geosystems* **12**, 7 (2011). |
| **7** | Gurevitch, E. L., Heunemann, C., Rad'ko, V., Westphal, M., Bachtadse, V., Pozzi, J. P., & Feinberg, H. Palaeomagnetism and magnetostratigraphy of the Permian–Triassic northwest central Siberian Trap Basalts. *Tectonophysics* **379**, 211-226 (2004). |
| **4, 8, 10** | Heunemann, C., Krása, D., Soffel, H. C., Gurevitch, E., & Bachtadse, V. Directions and intensities of the Earth’s magnetic field during a reversal: results from the Permo-Triassic Siberian trap basalts, Russia. *Earth and Planetary Science Letters* **218**, 197-213 (2004). |
| **1, 9** | Kravchinsky, V. A., Konstantinov, K. M., Courtillot, V., Savrasov, J. I., Valet, J. P., Cherniy, S. D., Mishenin, S. G., & Parasotka, B. S. Palaeomagnetism of East Siberian traps and kimberlites: two new poles and palaeogeographic reconstructions at about 360 and 250 Ma. *Geophysical Journal International* **148**, 1-33 (2002). |
| **3** | Latyshev, A. V., Veselovskiy, R. V., & Ivanov, A. V. Paleomagnetism of the Permian-Triassic intrusions from the Tunguska syncline and the  Angara-Taseeva depression, Siberian Traps Large Igneous Province: Evidence of contrasting styles of magmatism. *Tectonophysics*, **723**, 41-55 (2018). |
| **14** | Miguez, M., Dopico, C. I. M., Rapalini, A. E., Luppo, T., & Luchi, M. L. Paleomagnetism of a Permo-Triassic bimodal dike swarm of northern Patagonia. *In*: *Latinmag Letters* **6**, 1-6 (2016). |
| **5** | Pavlov, V. E., Fluteau, F., Veselovskiy, R. V., Fetisova, A. M., & Latyshev, A. V. Secular geomagnetic variations and volcanic pulses in the Permian-Triassic traps of the Norilsk and Maimecha-Kotui provinces. Izvestiya, *Physics of the Solid Earth* **47**, 402 (2011). |
| **13** | Tomezzoli, R. N., Saint Pierre, T., & Valenzuela, C. New palaeomagnetic results from Late Paleozoic volcanic units along the western Gondwana margin in La Pampa, Argentina. *Earth, Planets and Space* **61**, 183-189 (2009). |
| **11** | Van Der Voo, R., Wu, F., Zhongmin, W., Dongwoo, S., Peacor, D. R., & Qizhong, L. Paleomagnetism and electron microscopy of the Emeishan Basalts, Yunnan, China. *Tectonophysics*, **221**, 367-379 (1993). |
| **2, 6** | Veselovskiy, R. V., Konstantinov, K. M., Latyshev, A. V., & Fetisova, A. M. Paleomagnetism of the trap intrusive bodies in arctic Siberia: Geological and methodical implications. *Izvestiya, Physics of the Solid Earth* **48**, 738-750 (2012). |
| **12** | Yokoyama, E., Brandt, D., Tohver, E., & Trindade, R. I. Palaeomagnetism of the Permo-Triassic Araguainha impact structure (Central Brazil) and implications for Pangean reconstructions. *Geophysical Journal International* **198**, 154-163 (2014). |
